# Repeated long-distance dispersal and convergent evolution in hazel

**DOI:** 10.1038/s41598-019-52403-2

**Published:** 2019-11-05

**Authors:** Andrew J. Helmstetter, Richard J. A. Buggs, Stuart J. Lucas

**Affiliations:** 1 Jodrell Laboratory, Royal Botanic Gardens, Kew, TW9 3AB Richmond, UK; 2 Institut de Recherche pour le Développement (IRD), UMR-DIADE, BP 64501, 34394 Montpellier, France; 3 School of Biological and Chemical Sciences, Queen Mary University of London, London, E1 4NS UK; 4 Sabanci University Nanotechnology Research and Application Center (SUNUM), Sabanci University, Orhanlı, 34956 Tuzla, Istanbul Turkey

**Keywords:** Biogeography, Phylogenetics

## Abstract

Closely related species with a worldwide distribution provide an opportunity to understand evolutionary and biogeographic processes at a global scale. Hazel (*Corylus*) is an economically important genus of tree and shrub species found in temperate regions of Asia, North America and Europe. Here we use multiple nuclear and chloroplast loci to estimate a time-calibrated phylogenetic tree of the genus *Corylus*. We model the biogeographic history of this group and the evolutionary history of tree and shrub form. We estimate that multiple *Corylus* lineages dispersed long distances between Europe and Asia and colonised North America from Asia in multiple independent events. The geographic distribution of tree versus shrub form of species appears to be the result of 4–5 instances of convergent evolution in the past 25 million years. We find extensive discordance between our nuclear and chloroplast trees and potential evidence for chloroplast capture in species with overlapping ranges, suggestive of past introgression. The important crop species *C*. *avellana* is estimated to be closely related to *C*. *maxima*, *C*. *heterophylla* var. *thunbergii* and the Colurnae subsection. Our study provides a new phylogenetic hypothesis or *Corylus* and reveals how long-distance dispersal can shape the distribution of biodiversity in temperate plants.

## Introduction

In order to fully utilise the genetic potential of a cosmopolitan group of species we need to understand its evolutionary history and the biogeographic processes that shape its global patterns of diversity. When multiple species within a genus are present in the same geographic area, their distribution may be the result of speciation within the area, separate colonisation events by species that have originated elsewhere or a combination of the two. Ancestral biogeographic estimation methods can be used to model these historical patterns and typically separate cladogenesis into three types (summarised here^1^): where the new species occupy the same range as their ancestors, where a new species occupies a subset of the ancestral range or where speciation occurs as a result of a vicariance event (i.e. the splitting of an existing species range). Commonly used models such as Dispersal-Extinction-Cladogenesis (DEC)^2^ or Dispersal-Vicariance (DIVA)^3^ can model some or all of these types of cladogenesis.

In addition, long distance dispersal events can provide sufficient isolation from the ancestral population for a new lineage to diverge, potentially leading to speciation. Such founder-event speciation^4,5^ (which is not necessarily the result of founder effects) may play an important role in generating species diversity, especially in clades that are present worldwide, having presumably spread far from a single point of origin. Recent advances in ancestral range estimation methods have allowed the inclusion of this founder-event speciation into commonly used models^1^. Additionally, anagenetic events (range expansion and range contraction) can affect the geographic distribution of diversity and are also included in these models. Here, we investigate the relative importance of different cladogenetic and anagenetic events in shaping the worldwide distribution of diversity and growth form in the genus *Corylus*.

**Figure 1:**
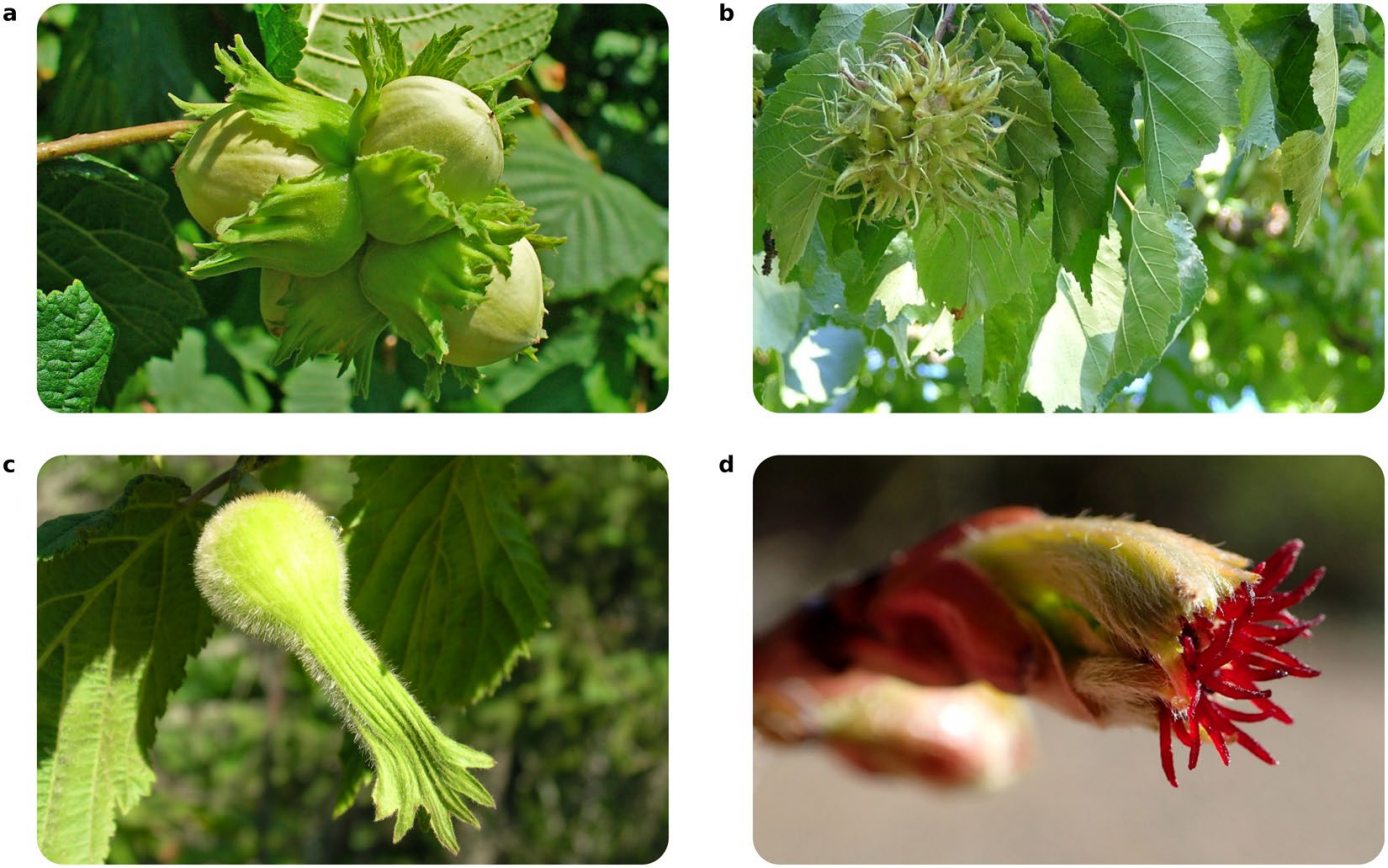
Four species in the genus *Corylus*. (**a**) *C*. *avellana*, the economically important common hazel found across Europe and into western Asia, (**b**) *C*. *colurna*, the Turkish hazel found in The Caucasus & Balkans, (**c**) the beaked hazel, *C*. *cornuta*, native to North America and (**d**) a flower of *C*. *sieboldiana*, the Japanese hazel. Photos taken from www.wikimedia.org with credit to (**a**) H. Zell (Published under the following license: https://creativecommons.org/licenses/by-sa/3.0/deed. Minor changes were made.), (**b**) Lottis 80 (Published under https://creativecommons.org/licenses/by-sa/3.0/deed. Minor changes were made.), (**c**) Superior National Forest (Published under https://creativecommons.org/licenses/by/2.0/deed.en. Minor changes were made.) and (**d**) Krzysztof Ziarnek (Published under https://creativecommons.org/licenses/by-sa/4.0/deed.en. Minor changes were made).

Members of the genus *Corylus* L. (Betulaceae), commonly known as hazel, are an economically important group of between 9–25 species depending on authority^6–8^ that are found in temperate regions of Asia, Europe and North America. Some of the diversity in reproductive structures is shown in Fig. 1. Species of *Corylus* are deciduous, monoecious trees and shrubs that produce hazelnuts, an important source of food for animals that is also cultivated extensively for human consumption. The hazelnut of *Corylus avellana* is the fifth most produced tree nut crop in the world and in Turkey, where approximately 70% of production is located^9^, more than one million tons of hazelnut are produced per year^6^. Characterising the evolutionary relationships between *C*. *avellana* and other *Corylus* species would enable the genetic resources present in closely related wild relatives to be used more effectively for crop improvement. Though *C*. *avellana* is by far the most important crop, nuts of all *Corylus* species are edible^6^. For example, nuts of *C*. *jacquemontii* & *C*. *colurna* are often consumed by local human populations^6^.

Numerous species of each growth form exist^6^ but where, when and how often growth form changed remains unknown. A change of growth form may lead to particular traits that are useful for cultivation. In areas where hazel is cultivated, tree form or shrub form may be preferable depending on the terrain and local agricultural practices^6^. Therefore, deciphering the genetic basis of these traits, and the transition between them, is important when developing new varieties. Understanding the evolutionary history of this genus is therefore important from both a scientific and economic standpoint.

Three previous studies have attempted to reconstruct the evolutionary history of *Corylus*. One used the Internal Transcribed Spacer (ITS) 1, ITS2 & 5.8S regions and morphology^10^ and another study used ITS1, ITS2 & 5.8S and maturase K (matK) regions^11^. The latest study^12^ used SNP data to infer phylogenetic trees. Regarding the biogeographic history of the group, Whitcher and Wen^10^ found that *Corylus* had dispersed to North America from East Asia and Europe independently. However, due to lack of resolution in phylogenetic trees, the order and frequency of major dispersal events remained uncertain. Most recently, Yang *et al*.^12^ reconstructed the biogeographic history of *Corylus* and also inferred this pattern, though varying topologies of inferred phylogenetic trees showed that *Corylus* lineages may have arrived in North America from East Asia twice and it is unclear which scenario is more likely. This indicates that uncertainty about the evolutionary and biogeographic history of the genus remains.

Understanding the fossil history of a group is critical if we are to provide a timescale to its evolutionary and biogeographic history. Known fossils relating to *Corylus* belong to stem group taxa^13^ or closely related extinct genera^14,15^. Fossilized pollen and mesofossils have been putatively classified as belonging to *Corylus* or other Betulaceae genera^16–18^, but more study using techniques such as electron microscopy must be done to confirm this to make them reliable enough for molecular dating^19^. We thus consider that there is an adequate fossil record available for preliminary dating of divergence events in this group.

Here we present new phylogenetic trees of the hazel genus, *Corylus*, inferred from chloroplast (cpDNA) and nuclear DNA (nrDNA) sequences available on GenBank. We estimate divergence times using calibration priors based on dated fossils, infer a biogeographic history for the group and uncover the evolutionary history of growth form in the genus. We ask three main questions: (i) how has the distribution of hazel changed through time? (ii) how important have different types of biogeographic events been in the history of *Corylus*? (iii) did tree form evolve once and spread or are different tree species the result of convergent evolution? We reveal evidence for convergent evolution in tree form and several major intercontinental dispersal events that have shaped the geographic distribution of hazel we see in nature today.

## Materials and Methods

### Phylogenetic inference

We collected sequences from 13 different loci across the 16 *Corylus* taxa that were available on Genbank (https://www.ncbi.nlm.nih.gov/genbank/ accessed Nov. 2016) and four outgroup species (*Alnus incana*, *Betula papyrifera*, *Carpinus japonica* and *Ostrya rehderiana*), selecting the longest sequence available per locus per taxon to maximize available information. These loci were; internal transcribed spacer 1 (ITS1), internal transcribed spacer 2 (ITS2) & 5.8S-rRNA, 5S-rRNA & Non-Transcribed Spacer (NTS), ribulose-1,5-bisphosphate carboxylase/oxygenase large subunit (*rbcL*), ATPase beta-subunit (*atpB-rbcL*) intergenic spacer, granule bound starch synthase (GBSSI), maturase K (*matK*), nitrate reductase (NIA), cytosolic phosphoglucose isomerase (*pgiC*), tRNA-His - photosystem II protein D1 intergenic spacer (*trnH-psbA*), ribosomal protein S16 (*RPS16*), ribosomal protein L16 (*RPL16*), tRNA-Leu (*trnL*) and trnL- tRNA-Phe (*trnL-trnF*) intergenic spacer. Sequences were aligned using the MAFFT 1.3.5 alignment plugin^20^ in GENEIOUS 10.0.6 (Biomatters).

To test if there were any erroneous sequences we built gene trees using the maximum-likelihood approach RAxML^21^ (v8.2.9). For each run we applied a GTRGAMMA model and used the ‘-f a’ option with 100 replicates for bootstrapping. We visually assessed each gene tree for unusually long branches or unlikely topologies (compared to preliminary trees in this study and those from previous studies) that may be indicative of error in the Genbank sequences. When a potentially incorrect sequence was identified, it was removed from the associated alignment for downstream phylogenetic analyses.

We used BEAST (v2.4.4)^22^, a Bayesian approach, to build time-calibrated trees. Partitions of nuclear and chloroplast loci were analysed separately so we could examine the differences between trees inferred from nuclear and plastid genomes. Sequence alignments were then imported into BEAUti (v2.4.4)^22^. A yule model was used as the tree prior. Each locus was treated as a partition to confer separate site and clock models while trees were linked across all partitions. We used the program modeltest-ng (v0.1.3) to determine the appropriate substitution models for each partition based on Bayesian Information Criteria (BIC). We added the +G (gamma rate heterogeneity) parameter to substitution models for *matk*, *rbcL* and *atpB-rbcL* to improve mixing after it was initially poor. Relaxed lognormal clock models were used for each partition to allow rates to vary among branches.

We took two separate time-calibration approaches. Trees were first calibrated with three lognormal priors, using published estimated ages of fossil records of *C*. *johnsonii*^13^, *Cranea*^14^ and *Palaeocarpinus*^15^. We used these to calibrate stem *Corylus*, stem Coryloideae and the root of our trees (Betulaceae) respectively. Lognormal calibration prior parameters were calculated as follows. The minimum age of the fossil’s estimated geological time period was used as a hard minimum. The M parameter (mean of the log-transformed distribution) was set so that the median of the lognormal distribution matched the middle point between the minimum and maximum age of the specified time period. The S parameter (standard deviation of the log-transformed distribution) was left at 1.25 so that a relatively long tail was present to act as a soft maximum rather than a hard maximum as in a uniform prior. When presenting results we refer to those from trees calibrated with lognormal priors, unless stated otherwise.

Second, we took a more conservative approach with uniform priors to examine how using a uniform probability distribution affects divergence times. We used the minimum age of each fossil as our hard minimum, and the 97.5% quantile for the lognormal prior on the root as our hard maximum in each case. We verified in preliminary runs that divergence times were not approaching the hard maximums of the uniform priors so as to ensure they were appropriate and not biasing our runs. Calibration parameters are shown in Table 1 and further details on fossils used and their inferred geological time periods can be found in Supplementary Appendix S1.

**Table 1:** Calibration parameters for tree inference.

Taxon	Lognormal prior			Uniform	
	Offset	M	S	Min.	Max.
Betulaceae	59.8	6.3	1.25	59.8	93.2
Stem Coryloideae	56	3.5	1.25	56	93.2
Stem *Corylus*	49	1	1.25	49	93.2

Offset is the hard minimum age for the lognormal prior. M and S are the mean and standard deviation of the log-transformed lognormal distribution respectively. Min and max represent the lower and upper cut-offs of the uniform prior distribution.

For the cpDNA and nrDNA trees we ran BEAST 2.4.4^11^ for 1.0 × 10^8^ and 5.0 × 10^7^ generations respectively, sampling runs to produce a posterior distribution of 10,000 trees. Two additional runs were conducted in each case and compared to the initial run to ensure stationarity had been reached at the same point across runs. We examined combined logfiles in TRACER 1.6^23^ to ensure stationarity had been reached (ESS greater than 100) for all parameters. Trees from these three runs were combined using LOGCOMBINER^22^ and the relevant proportion of burn-in was discarded. TREEANNOTATOR^22^ was then used to infer a maximum clade credibility (MCC) tree from the posterior distribution of combined trees, keeping target heights.

### Growth form

We used the ace function in the R package ‘ape’^24^ to reconstruct the evolution of growth form on nrDNA and cpDNA trees. We then used likelihood ratio tests to compare whether a model with equal transition rates (ER) or an all-rates-different (ARD) model fit better. We first ran models using members of the *Corylus* genus only and then conducted a further set of runs that included members of the two closest outgroup genera, *Ostrya* and *Carpinus*, to understand how the inclusion of these tree form taxa affected the root state of *Corylus*. We used data from Whitcher and Wen^10^ and Molnar^6^ to classify each species as “tree form” or “shrub form”. Species that are thought to be small trees or shrubs (such as *C*. *yunnanensis*), are classified as shrub form.

### Geographic data

Native geographic ranges were taken from Molnar^6^ where available. If no range information was available we examined records from the Global Biodiversity Information Facility (http://www.gbif.org/) and assigned taxa to regions based on occurrences. We checked these data and removed clearly erroneous entries and museum/cultivated individuals. For the differences in range between *C*. *sieboldiana* and *C*. *sieboldiana* var. *mandshurica* we used data from Chang *et al*.^25^. In the case of *Corylus maxima*, we used information from the IUCN red list of threatened species (http://www.iucnredlist.org/). We assigned each taxon to one or more geographic regions from Western Europe (W), The Caucasus & Balkans (T), North America (A), Japan & Korea (North and South) (J), China (C), Mongolia (M), Eastern Russia (E) and India & Nepal (I).

### Ancestral range estimation

We used the R package ‘BioGeoBEARS’^1^ to estimate ancestral ranges in *Corylus*. This package allows for inferences using three biogeographic models almost identical to the DEC model^2^, the DIVA model^3^ and the Bayesian Inference of Historical Biogeography for Discrete Areas (BayArea) model^26^. ‘BioGeoBEARS’ allowed us to estimate the importance of founder events in the biogeographic history of a group by introducing a new parameter (j) for jump dispersal (founder-event speciation). We performed two sets of analyses, the first where there were no restrictions on area combinations and the second where an “areas-allowed” matrix was used (Supplementary Table S1); this matrix summarises the geographical connectedness of different areas (including Eastern Russia & North America to incorporate a late tertiary Bering land bridge^27^) and this information is used to restrict area combinations. For each analysis set we ran all three models, with and without a jump dispersal parameter, where maximum number of areas per range was set to four (maximum observed in extant species) or eight (total number of areas). Models were compared using AICc to identify which were better supported by the data.

We also implemented Biogeographical Stochastic Mapping (BSM)^28^ in order to determine the frequency of biogeographic events in our models. This approach uses a given model to simulate the timing and location of all events on a tree. The biogeographic events this approach assesses are: within-area speciation - where speciation is within a single area and the range of both daughter nodes is identical to the parent node, within-area subset speciation - where speciation is within a range and the range of one daughter remains the same while the other is a single area subset of the parent node range, a vicariance event - where daughter nodes occupy non-overlapping subsets of the ancestral range, founder-event speciation - where a new species occupies an area unoccupied by its parent, range expansion and range contraction (summarized in^28^). We ran 500 simulations in order to determine the frequency of each of these types of biogeographic event using the best-fitting models for each tree, with and without jump dispersal.

## Results

### Phylogenetic inference

We collated data from four outgroup taxa and 16 *Corylus* taxa. Sequences from 13 partitions, consisting of eight chloroplast loci and five nuclear loci, were downloaded from Genbank. Sequences for each taxon were taken from five different sources on average (min: 2, max: 7) which will reduce any error caused by incorrectly labelled or identified GenBank sequences because identification mistakes are unlikely to be shared among sources. Furthermore, almost half (~44%) of sequences had a voucher associated, which suggests that these samples are reliable.

We further assessed potential error in our sequence data by building gene trees (Appendix S2) for each partition. Individual gene trees had varying levels of phylogenetic signal (see supplementary data) and bootstrap support was generally low, probably due to the relatively small amount of sequence data in each partition. Close inspection revealed several instances where sequences had long terminal branches and unusual topological placement. We removed *C*. *heterophylla* from the *trnl-trnf* alignment and *C*. *sieboldiana* var. *mandshurica* from our 5S-rRNA alignment. We initially removed *C*. *sieboldiana* from the *atpB-rbcl* alignment but subsequently found the entire alignment to converge very poorly during our Bayesian tree inference, so it was removed completely. The presence/absence of each locus for each species can be found in Supplementary Table S2 and the accession numbers of all sequences used can be found in Supplementary Table S3.

**Figure 2:**
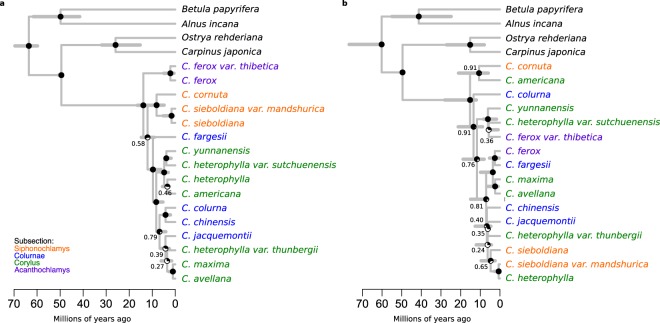
Time-calibrated maximum clade credibility trees constructed using (**a**) nuclear DNA and (**b**) chloroplast DNA with lognormal calibration priors. Node bars show 95% highest posterior density node ages. Posterior probability (PP) values are represented as pie charts on each node. Exact probability values are shown adjacent to nodes with PP < 0.95. Colours of taxa names indicate which subsection each species belongs to^6,10^.

Independent runs reached stationarity at the same point and after combination ESS values were greater than 200 for almost all parameters. Support for MCC trees within the genus was higher in the nrDNA (72% PP > 0.9) than cpDNA (61%) (Fig. 2a,b). Estimated crown node ages for *Corylus* ranged from 13.87–23.33 (10.26–32.47) Ma depending on calibrations and DNA dataset (Table 2). The nrDNA tree gives a noticeably younger age of *Corylus* than the cpDNA tree (Table 2).

**Table 2:** Node ages and minimum & maximum 95% Highest Posterior Density (HPD) heights taken from Maximum Clade Credibility (MCC) trees of all sequence data and calibration approaches.

Sequence	Prior	Clade	Node age (Ma)	95% HPD Min (Ma)	95% HPD Max (Ma)
Nuclear	Lognormal	Betulaceae	63.58	59.84	69.61
		Coryloideae	49.45	49.00	50.61
		Corylus	13.87	10.26	16.45
	Uniform	Betulaceae	84.77	65.98	93.18
		Coryloideae	55.70	49.00	63.21
		Corylus	19.31	11.49	19.65
Chloroplast	Lognormal	Betulaceae	60.21	59.84	76.74
		Coryloideae	49.57	49.00	50.62
		Corylus	15.19	12.01	28.10
	Uniform	Betulaceae	78.67	71.94	93.20
		Coryloideae	52.01	49.00	58.02
		Corylus	23.33	15.08	32.47

The recovered topologies were identical among different calibration approaches (Fig. 2; Supplementary Fig. S1). They revealed strong support of non-monophyly of varieties of *C*. *heterophylla* in both trees and *C*. *sieboldiana*/*C*. *ferox* in the cpDNA tree only. The topologies of the nrDNA (Fig. 2a) and cpDNA (Fig. 2b) trees differed extensively from each other. Some of these differences could be related to low support values but a number are well-supported incongruences. Most conspicuously, the two species found in North America (*C*. *cornuta* & *C*. *americana*) formed a sister pair with strong support in the cpDNA tree but were distantly related in the nrDNA tree. A clade containing *C*. *yunnanensis*, *C*. *ferox* var. *thibetica* and *C*. *heterophylla* var. *sutchuenensis* had strong support in the cpDNA tree (Fig. 2b) while in the nrDNA tree (Fig. 2a) *C*. *ferox* var. *thibetica* was sister to *C*. *ferox*. Similarly, *C*. *heterophylla* and *C*. *sieboldiana* var. *mandshurica* formed a sister pair in the cpDNA tree while in the nrDNA tree *C*. *sieboldiana* was more closely related to its conspecific *C*. *sieboldiana* var. *mandshurica* and *C*. *cornuta*. The cpDNA and nrDNA loci sets supporting these differences all came from multiple sources (Supplementary Table S3) indicating, along with our gene tree analysis, that misidentification was likely not the source of these incongruences. *Corylus avellana* and *C*. *maxima* were sister taxa in both trees.

### Ancestral range estimation

**Figure 3:**
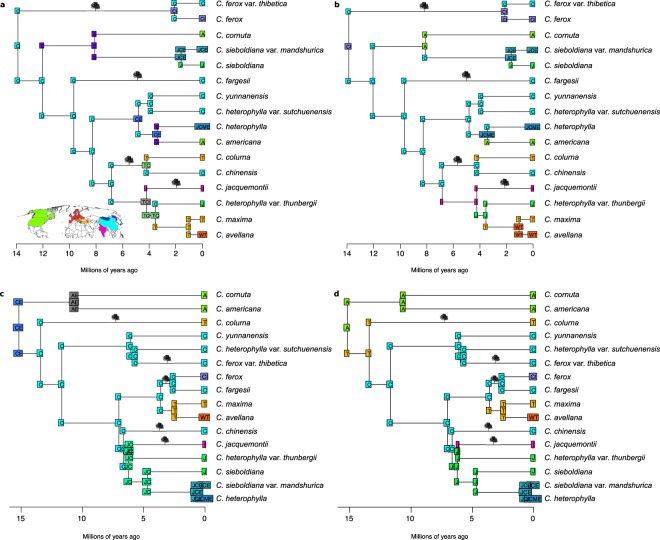
Ancestral range estimation using the best fitting models with and without jump dispersal for the nrDNA tree and cpDNA tree. For the nrDNA tree these models were (**a**) a Dispersal-Extinction-Cladogenesis (DEC) model and (**b**) a DEC model with the jump dispersal parameter (DEC + j). For the cpDNA tree models were (**c**) BayArea model and (**d**) a BayArea model with a jump dispersal parameter (BayArea + j). All models shown included an “areas-allowed” matrix. Nodes represent the ranges immediately before cladogenesis and corner labels show ranges immediately after. Tree silhouettes indicate the branches on which tree form likely evolved as estimated in ancestral state reconstructions. The inset world map shows an approximation of the eight areas used in the ancestral range estimation. Tree silhouette image used has been published under the license CC0 1.0 Universal (CC0 1.0) Public Domain Dedication.

Model selection revealed that the DEC + j model with an “areas-allowed” matrix fit the data best for the nrDNA tree (Fig. 3b; Supplementary Table S4) and the BayArea + j model with an “areas-allowed” matrix fit best for the cpDNA tree (Fig. 3d; Supplementary Table S4). In the nrDNA model *Corylus* most likely originated in east Asia, in a range containing China and India & Nepal while in the cpDNA model the most likely root state was only North America. Due to concerns about the validity of models with the jump dispersal parameter^29^ we also present the best fitting models without this parameter. In the DEC nrDNA model (Fig. 3a) China is the root state while it is China & Eastern Russia in the cpDNA BayArea model (Fig. 3c).

**Figure 4:**
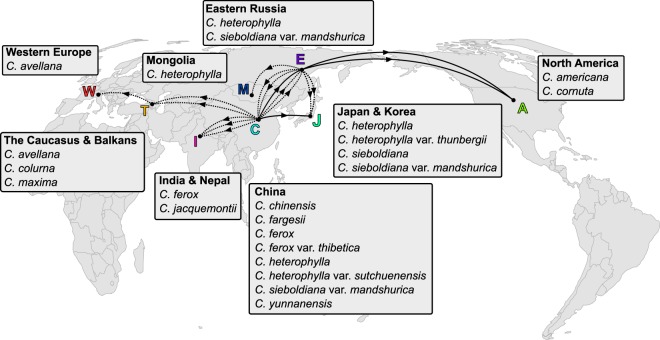
Modelled dispersal patterns of *Corylus* lineages shown on a world map. Ancestral ranges are taken from the most probable states of the nrDNA DEC model depicted in Fig. 3a. The species currently occurring in each region are labelled in adjacent boxes. Arrows on lines represent the direction of lineage dispersal. Solid lines represent a complete range shift where the ancestral region is no longer part of the lineage’s range. Dotted lines represent lineages that spread to a new region while also remaining in their ancestral region. There may be multiple dotted lines per lineage. The shape of the lines between regions does not represent the dispersal path. Vicariance events and range contractions (not inferred) are not represented on the figure.

Much of the speciation in the genus appears to have taken place in east Asia, particularly in China. This was frequently followed by major dispersal events elsewhere (Figs. 3, 4). Both nrDNA models estimate two long distance dispersal events from east Asia to North America (Fig. 3a,b). The earliest of these events occurred before (Fig. 3b) or after (Fig. 3a) the divergence of *C*. *sieboldiana* and *C*. *cornuta*, around 8 million years ago. A separate dispersal event from east Asia to North America was estimated in the *C*. *americana* lineage, less than 4 million years ago. *Corylus* dispersed from east Asia to The Caucasus & Balkans in two events in the nrDNA model. One of these preceded a single dispersal event to Western Europe that led to *C*. *avellana*.

The topology of the cpDNA tree was different to that of the nrDNA tree and this led to markedly different biogeographic histories. The principal differences related to the role of North America - just a single dispersal event to North America from east Asia is estimated in the cpDNA BayArea model. Likewise, the BayArea + j model suggested *Corylus* originated in North America and soon after dispersed to The Caucasus & Balkans. There were some similarities between the different datasets. For example, most of the diversification appears to have taken place in east Asia and The Caucasus & Balkans was colonised twice in all models.

In the best-fitting models without the jump dispersal parameter (Fig. 3a,c) we find a considerable increase in the number of times that Eastern Russia is part of an ancestral range. One major source of this increase is the movement of lineages from East Asia to North America, which go through Eastern Russia as this is the only area that connects the two continents in our models.

The “areas-allowed” matrix restricts the possible combinations of areas that can make up an ancestral range. However, when this was not included in the nrDNA DEC + j model, we found ancestral range estimations were little affected - additional areas were added to ancestral ranges in a small number of cases (Supplementary Fig S2a). However, some of these ranges included both North America and east Asian areas, which is geographically implausible. In the cpDNA BayArea + j model, most likely ranges did not differ from the model that included the “areas-allowed” matrix (Supplementary Fig. S2b). We also assessed the effect of removing the limit to the maximum number of areas for the two best-fitting models and found that the most probable states did not change.

We examined the frequency of the different types of biogeographic events in the best-fitting models using BSM analyses. Range expansion events were common, around half of all events, in both best fitting models without the jump dispersal parameter (Table 3). Within-area speciation was slightly more frequent in the BayArea model for the cpDNA tree while vicariance and the two types of within-area speciation were estimated to occur at relatively low levels in the DEC model for the nrDNA tree. We found that founder-event speciation was the most common event type in the nrDNA DEC + j model and made up approximately 33% of events compared to 28% in the cpDNA model (Table 3). Within-area speciation was the most common event type in the cpDNA BayArea + j model (47%). Range expansion events were relatively frequent in both + j models, while other event types were relatively rare. The frequency of within-area speciation was noticeably higher in China than any other area across all models.

**Table 3:** Results from Biogeographic Stochastic Mapping conducted on the set of best-fitting models for each tree detailing the mean and standard deviations of the frequency of each biogeographic event type after 500 simulations. Range contractions are not considered.

Model	Value	Founder event	Range expansion	WASS	Vicariance	WAS	Total events
nrDNA DEC	means	0	19.01	5.58	3.7	5.72	34.01
	stdevs	0	2.36	1.82	1.47	1.75	2.36
cpDNA BayArea	means	0	14.01	0	0	15	29.01
	stdevs	0	2.17	0	0	0	2.17
nrDNA DEC + j	means	7.18	6.98	3.91	0.97	2.95	21.98
	stdevs	1.38	0.96	1.56	0.95	1.4	0.96
cpDNA BayArea + j	means	5.54	5.01	0	0	9.46	20.01
	stdevs	0.71	0.077	0	0	0.71	0.077

Acronyms are used for within-area speciation (WAS) and within-area subset speciation (WASS).

### Growth form

The root state of the genus was most likely shrub form, although the probability of this state across all trees and models ranged from 50–58% (Supplementary Fig. S3). Probabilities at the root node were almost identical regardless of whether outgroups were included (Supplementary Fig. S3). If we assume the root state to be shrub form, ancestral state reconstructions using the nrDNA tree estimated four independent occasions during which tree form evolved (Fig. 3a,b; Supplementary Fig. S3). Matching the regions of the tree where we estimate tree form evolved to estimated ancestral ranges indicates that this trait originated three times in ranges involving China and once in India & Nepal. The reconstructions using the cpDNA tree are far less clear (Supplementary Fig. S3), but show that tree form evolved in five separate clades, three times in China, once in India & Nepal (though Fig. 3c indicates this instance could also have been in China and Japan & Korea) and once in China or The Caucasus & Balkans (Fig. 3c,d).

## Discussion

### Evolutionary History of *Corylus*

In this study we have improved our understanding of interspecific and intraspecific relationships in the genus *Corylus*. We used fossil calibrations to estimate that the genus likely originated in Miocene or Oligocene (Table 2). This large date range is due to difference in node ages between cpDNA and nrDNA and wider 95% HPD intervals for time-calibration based on uniform priors. Our results were similar to previous estimates of *Corylus* age in several studies^30–32^, but not as young those in Grimm and Renner^33^ who also took their data from GenBank but used different fossil calibrations.

Using sequence data from GenBank is a common practice (e.g.^33^) but may lead to the use of misidentified samples that could adversely affect phylogenetic tree inference^34^. Our initial dataset included up to 13 different loci from as many as seven different sources per taxon (Supplementary Table S3). We assessed each of our loci by inspecting gene trees for any unusual topology or extreme branch lengths, removing several sequences and a partition in the process. Almost half of our sequences had vouchers associated and any errors in identification are unlikely to be shared by sequences from different sources. In the event of an error the other loci in the dataset will help to minimize the effect of an incorrectly labelled locus. Therefore we believe that the potential impact of misidentified GenBank sequences is minimal.

With the addition of novel nuclear and chloroplast loci, our trees broadly reflect the findings of earlier studies. Whitcher and Wen^10^ used ITS1, ITS2, 5.8S and morphology to infer a tree and classify the genus *Corylus* into four different groups, which we refer to throughout this study (see subsections in Fig. 2). We found several similarities between their tree and our nrDNA tree. *Corylus ferox* made up a single group as the earliest diverging member of the genus and the subsection Siphonochlamys grouped *C*. *cornuta*, *C*. *sieboldiana* and *C*. *sieboldiana* var. *mandshurica*. Well-supported differences between the two phylogenetic hypotheses were largely found within these subsections rather than between them. For example, we find that *C*. *colurna* is sister to *C*. *chinensis* in our nrDNA tree instead of *C*. *jacquemontii* and uncover a strongly-supported sister relationship between *C*. *yunnanensis* and *C*. *heterophylla* var. *sutchuenensis*. In a separate study, Erdoğan and Mehlenbacher^11^ highlighted three main groups based on a tree constructed from ITS, similar to those used here but not including a group for *C*. *ferox*, which was absent from the study. This study also included a tree based on the chloroplast marker *matK* that grouped species from North America together as in our cpDNA tree (Fig. 2b), corroborating our inference and providing additional evidence to support the idea that cpDNA and nrDNA have different histories in *Corylus*.

A recent study by Yang *et al*.^12^ used SNP data to infer the evolutionary history of *Corylus*. Their study included most of the species used here as well as *C*. *wangii* and *C*. *californica* but their dataset lacked *C*. *maxima* and the variety *C*. *heterophylla* var. *thunbergii*. While Yang *et al*.^12^ generated more sequence data they concatenated all SNPs into a single locus and appear not to have accounted for the lack of invariant sites, which is known to bias phylogenetic inference and overestimate branch lengths^35,36^. Furthermore, they attempted to partition their dataset with recombination tests and evidence for recombination was used as a reason to remove SNPs from the dataset, resulting in a different tree topology^12^. These recombination tests were likely inappropriate as they were conducted on the concatenation of SNPs rather than contiguous sequences. Yang *et al*.^12^ find clades similar to those found here and in Whitcher and Wen^10^ but we note that *C*. *avellana* is placed either closely related to the Colurnae subsection (as in our nrDNA tree) or the Corylus subsection depending on tree inference approach taken, casting doubt on the reliability of their inferences. Divergence events in *Corylus* were generally found to be older in Yang *et al*.^12^ than in our study and could be attributed to biases caused by inferring branch lengths with SNP data only. Another source of error in their time-calibration may have come from their use of normally distributed priors on node age. This approach that is typically reserved for secondary calibrations or geological events^37^ and may lead to less accurate node ages if used for fossil calibrations as in Yang *et al*.^12^.

### Implications for cultivation

Identifying and characterising the close relatives of the *C*. *avellana* may inform interspecific breeding programmes to improve this important crop plant. *Corylus maxima* is thought by some to belong to the species *C*. *avellana*^6^ and here we confirm a close evolutionary relationship. However, estimated divergence times were older than one might expect within a single species: node age was 1.05–5.15 Ma depending on the data used. Confidence intervals suggest that divergence may have occurred as recently as 0.31 Ma so the level of isolation between these two lineages remains uncertain. It may be that *C*. *avellana* and *C*. *maxima* do not form a clade with the rest of the Corylus subsection (Fig. 2a). Even when genomic data is used the placement of *C*. *avellana* remains unclear^12^. The relationships in this group would be worth clarifying to shed more light on the closest relatives of the European hazelnut. Previous studies^10,11^ found *C*. *heterophylla* to be closely related to *C*. *avellana*, however our nrDNA tree indicates that *C*. *heterophylla* var. *thunbergii* is a closer relative of *C*. *avellana* than other members of *C*. *heterophylla*. It has been suggested that *Corylus avellana* could be crossed with *C*. *heterophylla* for its cold hardiness and ability to produce nuts at a young age^6,38^, and grafting might also be considered. Little is known about the *thunbergii* variety so further study could investigate whether the variety shares these traits with *C*. *heterophylla* and if it possesses any other potentially beneficial traits. *Corylus jacquemontii* diverged from *C*. *avellana* about 4–6 Ma in the nrDNA trees though we know little about its ability to cross with *C*. *avellana*^38^ or the benefits it could provide. It would be valuable to investigate whether these two species can cross, as this could be used to introgress traits possessed by *C*. *jacquemontii* such as the capacity to grow at high altitudes (up to 3000 m) and avoid suckering^6^.

The non-monophyly of species like *C*. *heterophylla* highlights an important implication of our results - some sub-species or varieties may actually have different evolutionary origins compared to their assumed conspecifics and could therefore be considered different species. The variety *C*. *heterophylla* var. *thunbergii* is not placed close to other *C*. *heterophylla* varieties in our inferred trees, but this may be due to poor resolution. Similarly, *C*. *sieboldiana* and *C*. *sieboldiana* var. *mandshurica* are not monophyletic in our cpDNA trees. We suggest that results in this study and others^12^ could be used as a platform for a taxonomic revision of the genus *Corylus*, particularly given the unclear boundary between variety and species and the wide range in number of species currently recognised by different authorities^6–8^.

### Effect of the jump dispersal parameter on ancestral range estimation

We found that the addition of the jump dispersal parameter improved the likelihood of both models we tested. Several other studies using BioGeoBEARS have found evidence for the widespread importance of jump dispersal in ancestral biogeographic models in animals^39,40^ and plants^28^ including temperate angiosperm lineages^41–43^. It is worth noting that the frequency of types of biogeographic events heavily depends on the size of the areas chosen (e.g. within-area speciation is more likely to occur in large areas such as China) and the model used, for example the DEC model without jump dispersal inflates the frequency of other event types (Table 3).

A recent criticism of DEC + j^29^ suggested that this model should be treated with caution because of some undesirable and unintuitive effects of adding the jump dispersal parameter. Indeed, our results using this model include improbable long distance dispersal events from China to North America and back again in rapid succession (Fig. 3b). The estimation of North America as the root state in the cpDNA BayArea + j model seems that it could have been biased by the jump dispersal parameter, as the root state has been estimated as a range in Asia in all other trees (Fig. 3). Consequently, we also examined results from models that did not include the jump dispersal parameter (Figs 3a,c, 4), which appear more realistic in most cases.

### Diversification and dispersal out of Asia

Much of the past diversification in hazel takes place in China and perhaps as a consequence of this, many dispersal events originate there (Figs 3, 4). We also see an important role for Eastern Russia (Fig. 3a,c) as an origin point for long distance dispersal towards North America. In nrDNA biogeographic models, both with and without jump dispersal, we consistently recover multiple dispersal events to North America and The Caucasus & Balkans. All models highlight an important role for east Asia in the diversification of the genus, particularly within China.

The colonisations of North America by the ancestors of *C*. *cornuta* and *C*. *americana* (Fig. 3a) probably occurred via the Bering land bridge^27^. We estimated this repeated dispersal regardless of the model used with our nrDNA tree, indicating it is a reliable set of events and not model-dependent. The resulting disjunction has previously been inferred in plants^44,45^ as well as animals^46^. As in this study, Whitcher and Wen^10^ found that *C*. *cornuta* traversed the Bering land bridge, but our results disagree with their suggestion that *C*. *americana* dispersed to North America from Western Europe across the North Atlantic. These patterns of dispersal fit well with the idea that much of the world’s extant temperate forest flora originated in east Asia and dispersed out during the last 30 million years^47^. Colonisation via the Bering land bridge is thought to contribute to the widespread pattern of disjunct distributions of temperate forest groups between North America and East Asia^48^.

Our second question was: how important have different types of biogeographic events been in the history of *Corylus*? To answer this we assessed the frequency of different types of biogeographic event. Range expansion, within-area speciation and founder-event speciation were generally the most common across our analyses (Table 3) suggesting that these processes were important in the history of *Corylus*. The relative importance of these events varied between cpDNA and nrDNA models. For example, range expansion and founder event speciation were more frequent in the nrDNA models and within-area speciation was more common in the cpDNA models (Table 3). Our cpDNA tree infers close relationships between geographically proximate species such as *C*. *americana* and *C*. *cornuta* that are not found in the nrDNA tree and this may be a source of the increased frequency of within-area speciation recovered. Again, the proportion of events inferred using models with jump dispersal must be treated with caution, though there appears to be scope for this process being important in *Corylus*.

### Frequent and repeated long distance dispersal

Our biogeographic models estimated several long distance dispersal events – thousands of kilometres over land. In most of our biogeographic models, lineages repeatedly dispersed from east Asia to The Caucasus & Balkans (Figs 3, 4). These lineages almost certainly passed through the regions between these areas but the history of dispersal across central Asia and the Middle East is unclear. Few species currently inhabit these regions *– C*. *avellana* is known to occur as far as Iran and Lebanon^49^ and *C*. *jacquemontii* is found in Northern Pakistan and Northeast Afghanistan^6^. The rarity of species records within these regions means that including the area in a model would likely not provide more resolution at the cost of increased model complexity and computation time. Instead, we coded the “areas-allowed” matrix to allow ranges including China and The Caucasus & Balkans in order to reduce this complexity. Further work could aim to identify the role of Central Asia and the Middle East in the biogeographic history of *Corylus*.

It has been hypothesised that European hazel spread at a rate of 1,500 m per year from glacial refugia in southern Europe^50,51^. This suggests that overland dispersal between Asia and The Caucasus & Balkans in the timeframes indicated by our phylogenetic trees is not unreasonable. Hazelnuts can be dispersed by both mammals and birds^52^, which could have helped species travel over these long distances. Bird species have been known to disperse hazelnuts up to six kilometres^53^ and mammals such as *Sciurus carolinensis* can store nuts for winter across more than a hectare of land around the plant^54^, which may have facilitated past dispersal. Dispersal across water bodies is also a possibility as hazelnuts can float and survive up to 90 days^52^. This characteristic may have facilitated dispersal across water bodies such as the Korea Strait, which lies between Japan and Korea and likely had to be crossed by species found in Japan today such as *C*. *sieboldiana*. Our ancestral range estimations consider only extant taxa and future work should include information from the fossil record to expand upon these models when fossils from the Betulaceae can be placed accurately. Further work incorporating these fossils and their morphology into the *Corylus* phylogenetic tree will develop our knowledge of the historical biogeography of the genus.

### Evidence for past introgression

The two North American hazel species, *C*. *cornuta* and *C*. *americana* are distantly related in the nrDNA tree but sister species in the cpDNA tree. Their ranges overlap extensively, with *C*. *cornuta* slightly further to the North and West than *C*. *americana*^6^. This proximity may have allowed interspecific hybridisation between the two species, leading to chloroplast capture^55^, where the chloroplast genome of one species is entirely replaced with that of another after introgression. Exchange of cpDNA has been observed between both tree and shrub species in North America^56,57^ and between species in North America and East Asia^58^. This process could have been a source of the incongruence we observe between our cpDNA and nrDNA trees. Further work with high-throughput sequence data could shed light on history of gene flow between *C*. *cornuta* and *C*. *americana*. Experiments have shown that these species do not readily hybridise in experimental crosses^38^ but this does not exclude the possibility that they were less reproductively isolated in the past. *Corylus ferox* var. *thibetica* and *C*. *heterophylla* var. *sutchuenensis* are both found in the Guizhou and Hubei provinces of southern and central China^6^. Due to the prospect of introgression and differences to the nrDNA tree, we suggest that the cpDNA tree represents the history of the chloroplast in *Corylus* rather than the species in the genus. Observed non-monophyly of some species may also be the result of past hybridisation. Other potential sources of differentiation between cpDNA and nDNA may be different evolutionary rates among these two marker types^59^ and incomplete lineage sorting^60^. The latter may be more prominent in recently diverged taxa or trees inferred with slowly evolving markers. Further work is needed to assess the degree to which chloroplast capture has occurred, and then use this information to determine which taxa are true species and which are varieties.

### Growth form

Our final question was: did tree form evolve once in *Corylus* or are different tree species the result of convergent evolution? Our models showed multiple instances of convergent evolution in growth form across the genus. Even though many species of *Carpinus* and *Ostrya* are trees, our reconstructions both with and without outgroups estimate that shrub form is the most likely ancestral state of the *Corylus* genus. Taking this into account, tree form probably arose on four to five separate occasions across the genus. Comparisons with biogeographic models revealed that in some cases lineages dispersed from east Asia towards The Caucasus & Balkans after tree form evolved. Therefore, convergent evolution as well as long-distance dispersal appears to have led to the geographic distribution of *Corylus* trees. There are many reasons why tree form may have evolved^61^ and future work identifying shared ecological characteristics or constraints of the widespread tree species may shed light on the drivers behind the inferred convergence.

## Conclusion

Our new phylogenetic trees resolve previously ambiguous relationships in the genus while adding additional taxa. Fossil calibrations allow the estimation of a detailed temporal scale of diversification in hazel, with most divergence events taking place in the Miocene. In our models, hazel appears to have originated in east Asia, diversifying and spreading throughout this area while dispersing west to The Caucasus & Balkans/Western Europe and east to North America in multiple, independent events. Our biogeographic models suggest that long distance dispersal was an important process in the history of the *Corylus* genus and instrumental in generating the diversity observed today, especially outside of east Asia. We estimate that multiple cases of convergent evolution of tree form have occurred in hazel species, mostly in China. Our study provides a platform for further investigations into potential introgression among geographically connected species, taxonomic revision of current varieties and the use of crop wild relatives to improve hazelnut production.

## Supplementary information

Supplementary Materials

## Data Availability

All tree files and BioGeoBEARS results files have been deposited in Figshare 10.6084/m9.figshare.10007618.v1.
