# Supplementary material for: Repeated long-distance dispersal and convergent evolution in hazel
Source: Sci Rep. 2019 Nov 5;9:16016. doi: 10.1038/s41598-019-52403-2 (PMC6831691; doi:10.1038/s41598-019-52403-2)

ELECTRONIC SUPPLEMENTARY MATERIAL

Repeated long-distance dispersal and convergent evolution in hazel

Andrew J. Helmstetter[1,2], Richard J. A. Buggs, Stuart J. Lucas

[1] Jodrell Laboratory, Royal Botanic Gardens, Kew, Richmond, Surrey UK. TW9 3AB

[2] Institut de Recherche pour le Développement (IRD), UMR-DIADE, BP 64501, F-34394, Montpellier cedex 5, France

Email: andrew.j.helmstetter@gmail.com

Appendix S1

Fossil calibrations

1. Betulaceae. This calibration point is based on fossils of the extinct genus Palaeocarpinus P. R. Crane[1,2]. These were fossilized fruits and leaves from several species dated from 59.8-65.5 Ma[3]. Manchester et al. (2004) write "…it is likely that Palaeocarpinus-like plants were the forerunners of both Coryleae and Carpineae."

2. Stem Coryloidae The extinct genus Cranea[4] was dated to the late Palaeocene (56-59.2 Ma) based on infructescences and associated leaves. Manchester and Chen[4] placed this genus in the Coryloidae based on a variety of traits including pollen and leaf morphology. Manchester and Chen[4] write "According to the features presented by leaves and pollen, the extinct genus belongs within the Coryloideae."

3. Stem Corylus. Corylus johnsonii from the Middle Eocene Klondike Mountain Formation, 50–49 Ma[1]. This extinct species resembles three extant species C. ferox, C. heterophylla and C. wangii.

References

1. Pigg K. B., Manchester, S. R. & Wehr, W. C. Corylus, Carpinus, and Palaeocarpinus (Betulaceae) from the middle Eocene Klondike mountain and Allenby formations of northwestern North America. Int. J. Plant Sci. 164, 807–822 (2003)

2. Manchester, S. R., Pigg, K. B. & Crane, P. R. Palaeocarpinus dakotensis sp. n. (Betulaceae: Coryloideae) and associated staminate catkins, pollen, and leaves from the Paleocene of North Dakota. Int. J. Plant Sci. 165, 1135–1148 (2004)

3. Sauquet, H. et al. Testing the impact of calibration on molecular divergence times using a fossil-rich group: the case of Nothofagus (Fagales). Syst. Biol. 61, 289–313 (2012).

4. Manchester, S. R. & Chen, Z. D. A new genus of Coryloideae (Betulaceae) from the Paleocene of North America. Int. J. Plant Sci. 159, 522–532 (1998).

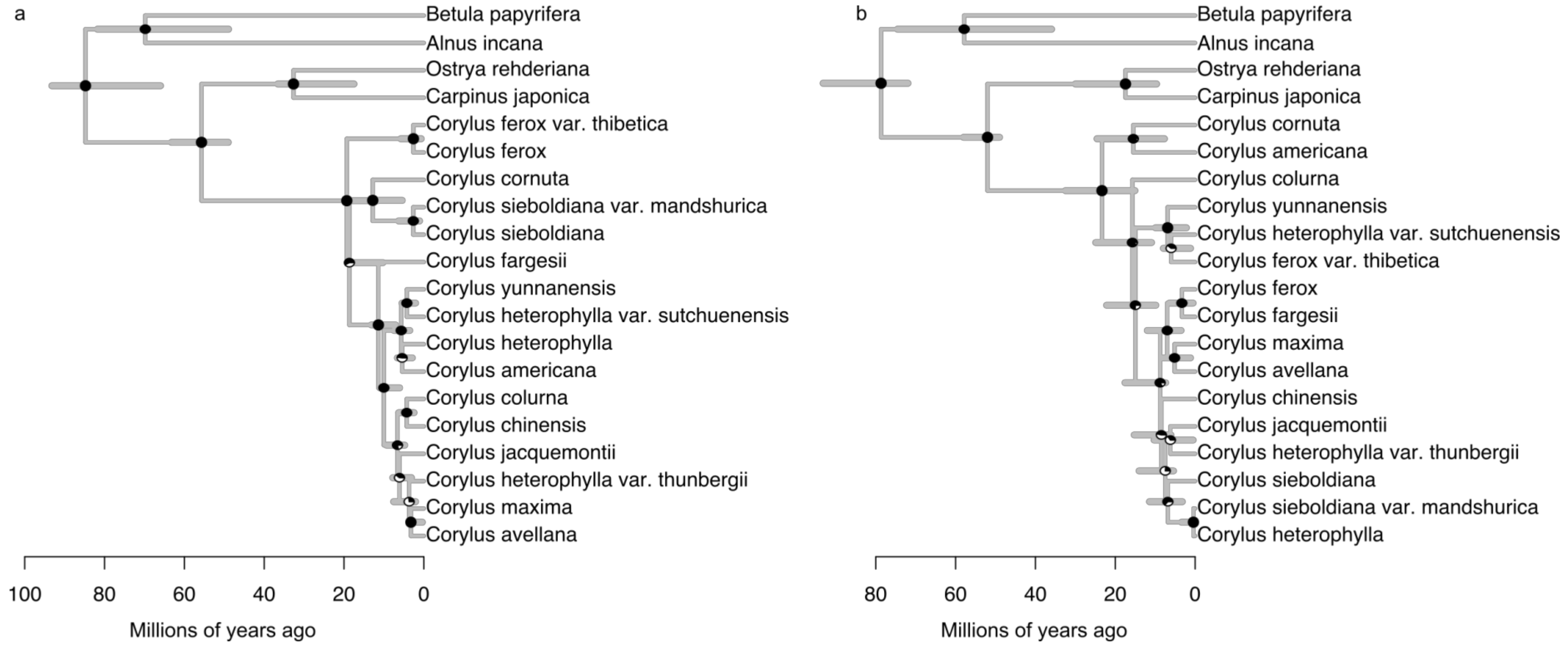

Figure S1 Time-calibrated maximum clade credibility trees constructed using (a) nuclear DNA and (b) chloroplast DNA only with uniform calibration priors. Node bars show 95% highest posterior density node ages. Posterior probability values are represented as pie charts on each node.

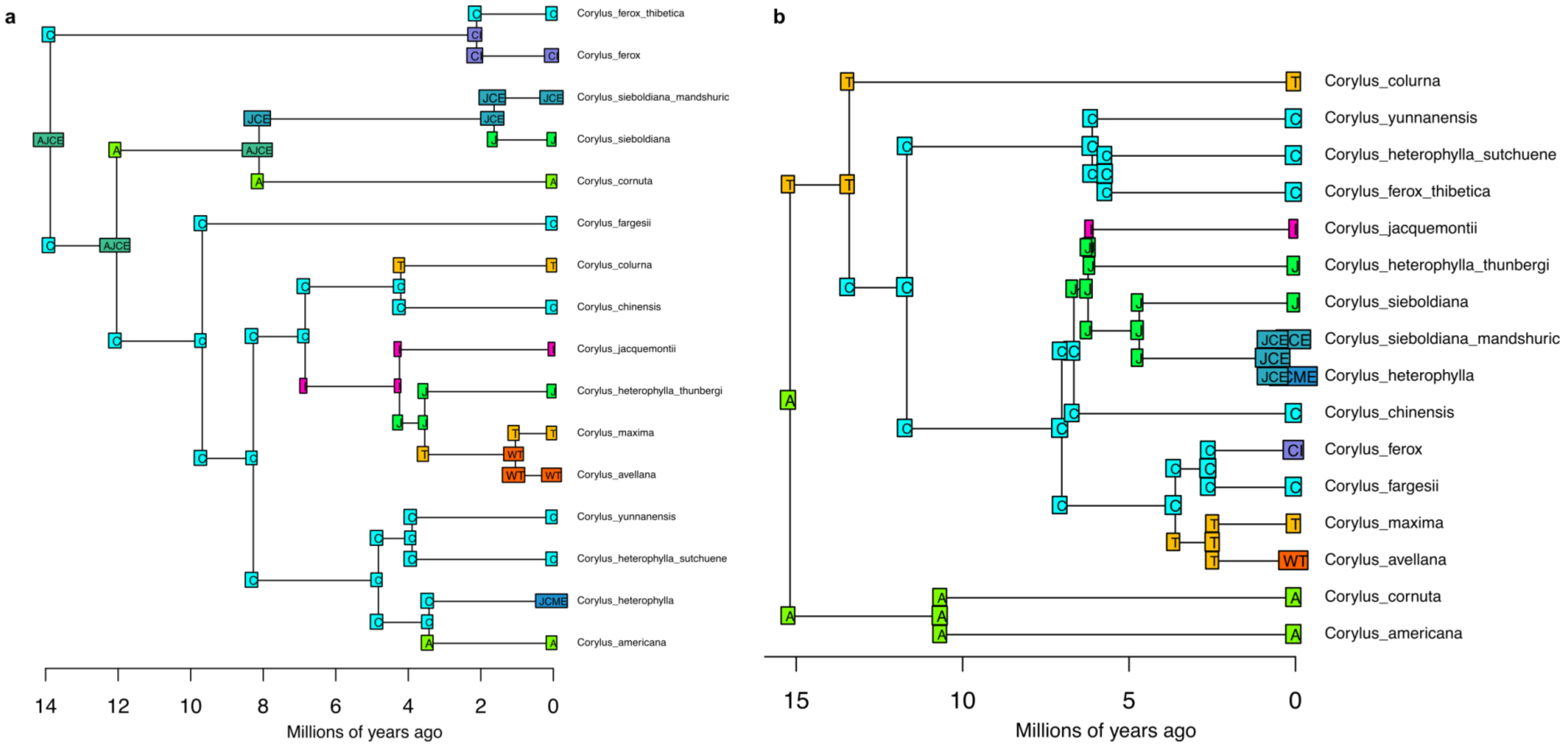

Figure S2 Ancestral range estimations using (a) nrDNA tree with DEC+j model with no range restrictions and (b) cpDNA tree with BayArea+j model with no range restrictions.

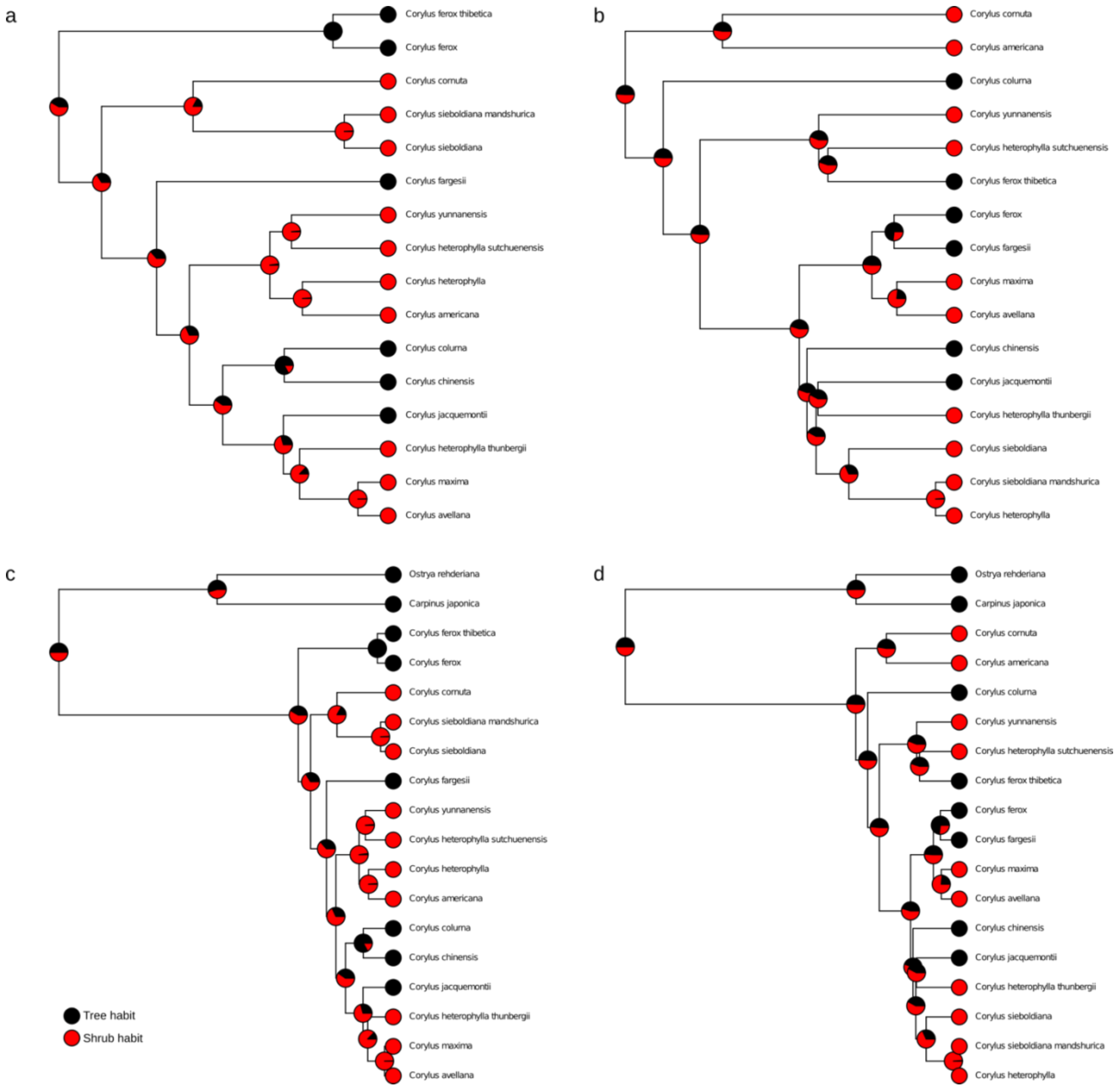

Figure S3 Ancestral state reconstructions of tree habit using nrDNA (a) & (c) and cpDNA (b) & (d) trees. No outgroup was included in (a) and (b) and two outgroup taxa were added for (c) and (d). Probabilities of ancestral states are shown on nodes of phylogenetic trees and current states are shown on the tips. In all cases, a likelihood ratio test of the more complex all-rates-different (ARD) model and the simpler equal-rates (ER) model supported the simpler model ($p > 0.05$).

Table S1 Areas allowed matrix used in BioGeoBEARS models

	W	T	A	J	C	M	E	I
W	1	1	0	0	0	0	0	0
T	1	1	0	0	1	0	0	1
A	0	0	1	0	0	0	1	0
J	0	0	0	1	1	1	1	0
C	0	1	0	1	1	1	1	1
M	0	0	0	1	1	1	1	0
E	0	0	1	1	1	1	1	0
I	0	1	0	0	1	0	0	1

Table S2 Presence/absence of loci for each taxon in this study. Dark grey cells represent sequences present with vouchers, light grey shows sequence are present without voucher (although many are clones or from chloroplast genomes) and white cells indicate absence.

Species	ITS1, ITS2 & 5.8S	5S & NTS	rbcL	atpB-rbcL	GBSSI	matK	NIA	pgiC	psbA-trnH	RPL16	RPS16	trnL-trnF	trnL
Alnus incana													
Betula papyrifera													
Carpinus japonica													
Corylus americana													
Corylus avellana													
Corylus chinensis													
Corylus colurna													
Corylus cornuta													
Corylus fargesii													
Corylus ferox													
Corylus ferox var. thibetica													
Corylus heterophylla var. sutchuenensis													
Corylus heterophylla var. thunbergii													
Corylus heterphylla													
Corylus jacquemontii													
Corylus sieboldiana var. mandshurica													
Corylus maxima													
Corylus sieboldiana													
Corylus yunnanensis													
Ostrya rehderiana													

Table S3 Accession numbers and descriptions of sequences taken from Genbank

Name	Accession	Description
Alnus_incana	FJ423677	Alnus incana atpB-rbcL intergenic spacer, partial sequence; and ribulose-1,5-bisphosphate carboxylase/oxygenase large subunit (rbcL) gene, partial cds; chloroplast
Alnus_incana	FJ423726	Alnus incana granule bound starch synthase (GBSSI) gene, exons 4, 5 and partial cds
Alnus_incana	M68868	Alnus incana 5S ribosomal RNA gene, complete sequence
Alnus_incana	AJ251665	Alnus incana 18S rRNA gene, 5.8S rRNA gene, 25S rRNA gene, internal transcribed spacer 1 (ITS1) and internal transcribed spacer 2 (ITS2)
Alnus_incana	KF419026	Alnus incana maturase K (matK) gene, partial cds; chloroplast
Alnus_incana	FJ041274	Alnus incana voucher CS82185 cytosolic phosphoglucose isomerase (pgiC) gene, exons 13 through 15 and partial cds
Alnus_incana	FJ011870	Alnus incana voucher CS82185 PsbA (psbA) gene, partial cds; psbA-trnH intergenic spacer, complete sequence; and tRNA-His (trnH) gene, partial sequence; chloroplast
Alnus_incana	X56618	A. incana rbcL gene for ribulose-biphosphate carboxylase (EC 4.1.1.39)
Alnus_incana	KP244625	Alnus incana voucher 065I0667 rps16 (rps16) gene, partial cds; and rps16-trnK intergenic spacer, partial sequence; chloroplast
Alnus_incana	FJ012048	Alnus incana voucher CS82185 tRNA-Leu (trnL) gene, partial sequence; trnL-trnF intergenic spacer, complete sequence; and tRNA-Phe (trnF) gene, partial sequence; chloroplast
Alnus_incana	FJ012048	Alnus incana voucher CS82185 tRNA-Leu (trnL) gene, partial sequence; trnL-trnF intergenic spacer, complete sequence; and tRNA-Phe (trnF) gene, partial sequence; chloroplast
Betula_papyrifera	FJ012006	Betula papyrifera voucher CS78048 rps16 gene, intron; chloroplast
Betula_papyrifera	FJ041284	Betula papyrifera voucher CS78048 cytosolic phosphoglucose isomerase (pgiC) gene, exons 13 through 15 and partial cds
Betula_papyrifera	X56617	B. papyrifera rbcL gene for ribulose-biphosphate carboxylase (EC 4.1.1.39)
Betula_papyrifera	AY372022	Betula papyrifera maturase K (matK) gene, complete cds; chloroplast
Betula_papyrifera	FJ011880	Betula papyrifera voucher CS78048 PsbA (psbA) gene, partial cds; psbA-trnH intergenic spacer, complete sequence; and tRNA-His (trnH) gene, partial sequence; chloroplast
Betula_papyrifera	M68869	Betula papyrifera 5S ribosomal RNA gene, complete sequence
Betula_papyrifera	FJ423732	Betula papyrifera granule bound starch synthase (GBSSI) gene, exons 4, 5 and partial cds
Betula_papyrifera	AF432067	Betula papyrifera internal transcribed spacer 1, partial sequence; 5.8S ribosomal RNA gene, complete sequence; and internal transcribed spacer 2, partial sequence
Betula_papyrifera	FJ423683	Betula papyrifera atpB-rbcL intergenic spacer, partial sequence; and ribulose-1,5-bisphosphate carboxylase/oxygenase large subunit (rbcL) gene, partial cds; chloroplast
Betula_papyrifera	FJ012058	Betula papyrifera voucher CS78048 tRNA-Leu (trnL) gene, partial sequence; trnL-trnF intergenic spacer, complete sequence; and tRNA-Phe (trnF) gene, partial sequence; chloroplast
Betula_papyrifera	KF418993	Betula papyrifera tRNA-Leu (trnL) gene, partial sequence; plastid

Carpinus_japonica	AB237199	Carpinus japonica chloroplast rps16 gene, partial sequence, isolate: AKUMA86
Carpinus_japonica	AJ784239	Carpinus japonica partial 5S rRNA gene, NTS and partial 5S rRNA gene, specimen voucher Forest 97-19 (MT)
Carpinus_japonica	FJ423692	Carpinus japonica granule bound starch synthase (GBSSI) gene, exons 4, 5 and partial cds
Carpinus_japonica	AF432035	Carpinus japonica internal transcribed spacer 1, partial sequence; 5.8S ribosomal RNA gene, complete sequence; and internal transcribed spacer 2, partial sequence
Carpinus_japonica	FJ011784	Carpinus japonica voucher A1034 maturaseK (matK) gene, partial cds; chloroplast
Carpinus_japonica	EU692813	Carpinus japonica clone c13 nitrate reductase gene, partial cds; and intron 3
Carpinus_japonica	FJ041211	Carpinus japonica voucher A1034 cytosolic phosphoglucose isomerase (pgiC) gene, exons 13 through 15 and partial cds
Carpinus_japonica	FJ011836	Carpinus japonica voucher A1034 PsbA (psbA) gene, partial cds; psbA-trnH intergenic spacer, complete sequence; and tRNA-His (trnH) gene, partial sequence; chloroplast
Carpinus_japonica	FJ423643	Carpinus japonica atpB-rbcL intergenic spacer, partial sequence; and ribulose-1,5-bisphosphate carboxylase/oxygenase large subunit (rbcL) gene, partial cds; chloroplast
Carpinus_japonica	FJ012015	Carpinus japonica voucher A1038 tRNA-Leu (trnL) gene, partial sequence; trnL-trnF intergenic spacer, complete sequence; and tRNA-Phe (trnF) gene, partial sequence; chloroplast
Carpinus_japonica	FJ012015	Carpinus japonica voucher A1038 tRNA-Leu (trnL) gene, partial sequence; trnL-trnF intergenic spacer, complete sequence; and tRNA-Phe (trnF) gene, partial sequence; chloroplast
Corylus_americana	FJ011972.1	Corylus americana voucher WN8564 rps16 gene, intron; chloroplast
Corylus_americana	KF418949.1	Corylus americana ribulose-1,5-bisphosphate carboxylase/oxygenase large subunit (rbcL) gene, partial cds; chloroplast
Corylus_americana	AY211448.1	Corylus americana chloroplast PsbA (psbA) gene, partial sequence; psbA-trnH intergenic spacer region, partial sequence; and tRNA-His (trnH) gene, partial sequence
Corylus_americana	FJ041239.1	Corylus americana voucher WN8564 cytosolic phosphoglucose isomerase (pgiC) gene, exons 13 through 15 and partial cds
Corylus_americana	EU692832.1	Corylus americana clone c22 nitrate reductase gene, partial cds; and intron 3
Corylus_americana	AY212001.1	Corylus americana maturase K (matK) gene, partial cds
Corylus_americana	FJ423703.1	Corylus americana granule bound starch synthase (GBSSI) gene, exons 4, 5 and partial cds
Corylus_americana	FJ423654.1	Corylus americana atpB-rbcL intergenic spacer, partial sequence; and ribulose-1,5-bisphosphate carboxylase/oxygenase large subunit (rbcL) gene, partial cds; chloroplast
Corylus_americana	AF254267.1	Corylus americana clone 1B 5S ribosomal RNA non-transcribed spacer
Corylus_americana	AF297349.1	Corylus americana clone Came3ITS internal transcribed spacer 1, partial sequence; 5.8S ribosomal RNA gene, complete sequence; and internal transcribed spacer 2, partial sequence
Corylus_americana	AY211417	Corylus americana chloroplast tRNA-Leu (trnL) gene, partial sequence; trnL-trnF intergenic spacer region, complete sequence; and trnF gene, partial sequence
Corylus_americana	AY211417	Corylus americana chloroplast tRNA-Leu (trnL) gene, partial sequence; trnL-trnF intergenic spacer region, complete sequence; and trnF gene, partial sequence
Corylus_americana	FJ011909	"Corylus americana voucher WN8564 ribosomal protein L16 (rpl16) gene, exons 1, 2 and partial cds; chloroplast"
Corylus_avellana	FJ011973.1	Corylus avellana voucher CS81224 rps16 gene, intron; chloroplast
Corylus_avellana	NC_031855	Corylus avellana chloroplast, complete genome

Corylus_avellana	FJ041240.1	Corylus avellana voucher CS81224 cytosolic phosphoglucose isomerase (pgiC) gene, exons 13, 14 and partial cds
Corylus_avellana	NC_031855	Corylus avellana chloroplast, complete genome
Corylus_avellana	FJ423704.1	Corylus avellana granule bound starch synthase (GBSSI) gene, exons 4, 5 and partial cds
Corylus_avellana	FJ423655.1	Corylus avellana atpB-rbcL intergenic spacer, partial sequence; and ribulose-1,5-bisphosphate carboxylase/oxygenase large subunit (rbcL) gene, partial cds; chloroplast
Corylus_avellana	AF254273.1	Corylus avellana clone 1A 5S ribosomal RNA non-transcribed spacer
Corylus_avellana	HQ442261.1	Corylus avellana isolate Ca38 18S ribosomal RNA gene, partial sequence; internal transcribed spacer 1, 5.8S ribosomal RNA gene, and internal transcribed spacer 2, complete sequence; and 28S ribosomal RNA gene, partial sequence
Corylus_avellana	NC_031855	Corylus avellana chloroplast, complete genome
Corylus_avellana	AY147072	Corylus avellana isolate T6 tRNA-Leu (trnL) gene, partial sequence; trnL-trnF intergenic spacer region, partial sequence; chloroplast gene for chloroplast product
Corylus_avellana	AY147072	Corylus avellana isolate T6 tRNA-Leu (trnL) gene, partial sequence; trnL-trnF intergenic spacer region, partial sequence; chloroplast gene for chloroplast product
Corylus_avellana	FJ011910	"Corylus avellana voucher CS81224 ribosomal protein L16 (rpl16) gene, exons 1, 2 and partial cds; chloroplast"
Corylus_chinensis	FJ011974.1	Corylus chinensis voucher Wen9026 rps16 gene, intron; chloroplast
Corylus_chinensis	KF418950.1	Corylus chinensis ribulose-1,5-bisphosphate carboxylase/oxygenase large subunit (rbcL) gene, partial cds; chloroplast
Corylus_chinensis	FJ011849.1	Corylus chinensis voucher Wen9026 PsbA (psbA) gene, partial cds; psbA-trnH intergenic spacer, complete sequence; and tRNA-His (trnH) gene, partial sequence; chloroplast
Corylus_chinensis	FJ041241.1	Corylus chinensis voucher Wen9026 cytosolic phosphoglucose isomerase (pgiC) gene, exons 13 through 15 and partial cds
Corylus_chinensis	AY212002.1	Corylus chinensis maturase K (matK) gene, partial cds
Corylus_chinensis	FJ423705.1	Corylus chinensis granule bound starch synthase (GBSSI) gene, exons 4, 5 and partial cds
Corylus_chinensis	FJ423656.1	Corylus chinensis atpB-rbcL intergenic spacer, partial sequence; and ribulose-1,5-bisphosphate carboxylase/oxygenase large subunit (rbcL) gene, partial cds; chloroplast
Corylus_chinensis	AF254300.1	Corylus chinensis clone 1A 5S ribosomal RNA non-transcribed spacer
Corylus_chinensis	AF297358.1	Corylus chinensis clone Cchi4ITS internal transcribed spacer 1, partial sequence; 5.8S ribosomal RNA gene, complete sequence; and internal transcribed spacer 2, partial sequence
Corylus_chinensis	AY211418	Corylus chinensis chloroplast tRNA-Leu (trnL) gene, partial sequence; trnL-trnF intergenic spacer region, complete sequence; and tRNA-Phe (trnF) gene, partial sequence
Corylus_chinensis	AY211418	Corylus chinensis chloroplast tRNA-Leu (trnL) gene, partial sequence; trnL-trnF intergenic spacer region, complete sequence; and tRNA-Phe (trnF) gene, partial sequence
Corylus_chinensis	FJ011911	"Corylus chinensis voucher Wen9026 ribosomal protein L16 (rpl16) gene, exon 2 and partial cds; chloroplast"
Corylus_colurna	FJ011975.1	Corylus colurna voucher CS96134 rps16 gene, intron; chloroplast
Corylus_colurna	KF418951.1	Corylus colurna ribulose-1,5-bisphosphate carboxylase/oxygenase large subunit (rbcL) gene, partial cds; chloroplast
Corylus_colurna	AF297384.1	Corylus colurna clone Ccol2matK maturase K (matK) gene, partial cds; chloroplast gene for chloroplast product
Corylus_colurna	FJ423706.1	Corylus colurna granule bound starch synthase (GBSSI) gene, exons 4, 5 and partial cds

Corylus_colurna	FJ423657.1	Corylus colurna atpB-rbcL intergenic spacer, partial sequence; and ribulose-1,5-bisphosphate carboxylase/oxygenase large subunit (rbcL) gene, partial cds; chloroplast
Corylus_colurna	AJ784235.1	Corylus colurna partial 5S rRNA gene, NTS and partial 5S rRNA gene, specimen voucher Forest 97-49 (MT)
Corylus_colurna	HQ442240.1	Corylus colurna isolate Ca15 18S ribosomal RNA gene, partial sequence; internal transcribed spacer 1, 5.8S ribosomal RNA gene, and internal transcribed spacer 2, complete sequence; and 28S ribosomal RNA gene, partial sequence
Corylus_colurna	FJ012028	Corylus colurna voucher CS96134 tRNA-Leu (trnL) gene, partial sequence; trnL-trnF intergenic spacer, complete sequence; and tRNA-Phe (trnF) gene, partial sequence; chloroplast
Corylus_colurna	FJ012028	Corylus colurna voucher CS96134 tRNA-Leu (trnL) gene, partial sequence; trnL-trnF intergenic spacer, complete sequence; and tRNA-Phe (trnF) gene, partial sequence; chloroplast
Corylus_colurna	FJ011912	"Corylus colurna voucher CS96134 ribosomal protein L16 (rpl16) gene, exons 1, 2 and partial cds; chloroplast"
Corylus_cornuta	X56619.1	C. cornuta rbcL gene for ribulose-biphosphate carboxylase (EC 4.1.1.39)
Corylus_cornuta	KP643275.1	Corylus cornuta voucher SNP_13_0405 psbA-trnH intergenic spacer, partial sequence; chloroplast
Corylus_cornuta	FJ041243.1	Corylus cornuta voucher CS96134 cytosolic phosphoglucose isomerase (pgiC) gene, exons 13, 14 and partial cds
Corylus_cornuta	EU692839.1	Corylus cornuta clone c19 nitrate reductase gene, partial cds; and intron 3
Corylus_cornuta	AY212003.1	Corylus cornuta maturase K (matK) gene, partial cds
Corylus_cornuta	AF254319	Corylus cornuta subsp. cornuta clone 1A 5S ribosomal RNA non-transcribed spacer
Corylus_cornuta	AF297338	Corylus cornuta clone Ccor1ITS internal transcribed spacer 1, partial sequence; 5.8S ribosomal RNA gene, complete sequence; and internal transcribed spacer 2, partial sequence
Corylus_cornuta	AY211419	Corylus cornuta chloroplast tRNA-Leu (trnL) gene, partial sequence; trnL-trnF intergenic spacer, complete sequence; and tRNA-Phe (trnF) gene, partial sequence
Corylus_cornuta	AY211419	Corylus cornuta chloroplast tRNA-Leu (trnL) gene, partial sequence; trnL-trnF intergenic spacer, complete sequence; and tRNA-Phe (trnF) gene, partial sequence
Corylus_fargesii	FJ011976.1	Corylus fargesii voucher UNA67862L rps16 gene, intron; chloroplast
Corylus_fargesii	NC_031854	Corylus fargesii chloroplast, complete genome
Corylus_fargesii	FJ041244.1	Corylus fargesii voucher UNA67862J cytosolic phosphoglucose isomerase (pgiC) gene, exons 13 through 15 and partial cds
Corylus_fargesii	EU692843.1	Corylus fargesii clone c28 nitrate reductase gene, partial cds; and intron 3
Corylus_fargesii	NC_031854	Corylus fargesii chloroplast, complete genome
Corylus_fargesii	FJ423707.1	Corylus fargesii granule bound starch synthase (GBSSI) gene, exons 4, 5 and partial cds
Corylus_fargesii	FJ423658.1	Corylus fargesii atpB-rbcL intergenic spacer, partial sequence; and ribulose-1,5-bisphosphate carboxylase/oxygenase large subunit (rbcL) gene, partial cds; chloroplast
Corylus_fargesii	FJ011741.1	Corylus fargesii voucher UNA67862L 18S ribosomal RNA gene, partial sequence; internal transcribed spacer 1, 5.8S ribosomal RNA gene, and internal transcribed spacer 2, complete sequence; and 26S ribosomal RNA gene, partial sequence
Corylus_fargesii	NC_031854	Corylus fargesii chloroplast, complete genome
Corylus_fargesii	FJ012029	Corylus fargesii voucher UNA67862L tRNA-Leu (trnL) gene, partial sequence; trnL-trnF intergenic spacer, complete sequence; and tRNA-Phe (trnF) gene, partial sequence; chloroplast
Corylus_fargesii	FJ012029	Corylus fargesii voucher UNA67862L tRNA-Leu (trnL) gene, partial sequence; trnL-trnF intergenic spacer, complete sequence; and tRNA-Phe (trnF) gene, partial sequence; chloroplast

Corylus_fargesii	FJ011913	"Corylus fargesii voucher UNA67862L ribosomal protein L16 (rpl16) gene, exons 1, 2 and partial cds; chloroplast"
Corylus_ferox	FJ011977.1	Corylus ferox voucher Wen9020 rps16 gene, intron; chloroplast
Corylus_ferox	FJ041246.1	Corylus ferox voucher Tibet810 cytosolic phosphoglucose isomerase (pgiC) gene, exons 13, 14 and partial cds
Corylus_ferox	FJ423708.1	Corylus ferox granule bound starch synthase (GBSSI) gene, exons 4, 5 and partial cds
Corylus_ferox	FJ423659.1	Corylus ferox atpB-rbcL intergenic spacer, partial sequence; and ribulose-1,5-bisphosphate carboxylase/oxygenase large subunit (rbcL) gene, partial cds; chloroplast
Corylus_ferox	AJ784233.1	Corylus ferox partial 5S rRNA gene, NTS and partial 5S rRNA gene, specimen voucher RBGE 19930413B
Corylus_ferox	FJ011743.1	Corylus ferox voucher Wen9020 18S ribosomal RNA gene, partial sequence; internal transcribed spacer 1, 5.8S ribosomal RNA gene, and internal transcribed spacer 2, complete sequence; and 26S ribosomal RNA gene, partial sequence
Corylus_ferox	FJ011800	Corylus ferox voucher Wen9020 maturaseK (matK) gene, partial cds; chloroplast
Corylus_ferox	FJ011852	Corylus ferox voucher Wen9020 PsbA (psbA) gene, partial cds; psbA-trnH intergenic spacer, complete sequence; and tRNA-His (trnH) gene, partial sequence; chloroplast
Corylus_ferox	FJ012030	Corylus ferox voucher Wen9020 trnL-trnF intergenic spacer and tRNA-Phe (trnF) gene, partial sequence; chloroplast
Corylus_ferox	FJ011914	"Corylus ferox voucher Wen9020 ribosomal protein L16 (rpl16) gene, exons 1, 2 and partial cds; chloroplast"
Corylus_ferox_thibetica	AF297391.1	Corylus ferox var. tibetica clone Cfer1matK maturase K (matK) gene, partial cds; chloroplast gene for chloroplast product
Corylus_ferox_thibetica	AF297361.1	Corylus ferox var. tibetica clone Cfer1ITS internal transcribed spacer 1, partial sequence; 5.8S ribosomal RNA gene, complete sequence; and internal transcribed spacer 2, partial sequence
Corylus_ferox_thibetica	FJ011983.1	Corylus ferox var. thibetica voucher Tibet1679 rps16 gene, intron; chloroplast
Corylus_ferox_thibetica	FJ011858.1	Corylus ferox var. thibetica voucher Tibet1679 PsbA (psbA) gene, partial cds; psbA-trnH intergenic spacer, complete sequence; and tRNA-His (trnH) gene, partial sequence; chloroplast
Corylus_ferox_thibetica	FJ041254.1	Corylus ferox var. thibetica voucher Tibet1679 cytosolic phosphoglucose isomerase (pgiC) gene, exons 13, 14 and partial cds
Corylus_ferox_thibetica	EU692855.1	Corylus ferox var. thibetica clone c1 nitrate reductase gene, partial cds; and intron 3
Corylus_ferox_thibetica	FJ423714.1	Corylus ferox var. thibetica granule bound starch synthase (GBSSI) gene, exons 4, 5 and partial cds
Corylus_ferox_thibetica	FJ423665.1	Corylus ferox var. thibetica atpB-rbcL intergenic spacer, partial sequence; and ribulose-1,5-bisphosphate carboxylase/oxygenase large subunit (rbcL) gene, partial cds; chloroplast
Corylus_ferox_thibetica	FJ012036	Corylus ferox var. thibetica voucher Tibet1679 trnL-trnF intergenic spacer and tRNA-Phe (trnF) gene, partial sequence; chloroplast
Corylus_ferox_thibetica	FJ011920	"Corylus ferox var. thibetica voucher Tibet1679 ribosomal protein L16 (rpl16) gene, exons 1, 2 and partial cds; chloroplast"
Corylus_heterophylla	FJ011978.1	Corylus heterophylla voucher Yoo s.n. rps16 gene, intron; chloroplast
Corylus_heterophylla	NC_031856	Corylus heterophylla chloroplast, complete genome
Corylus_heterophylla	FJ041248.1	Corylus heterophylla voucher Yoo s.n. cytosolic phosphoglucose isomerase (pgiC) gene, exons 13 through 15 and partial cds
Corylus_heterophylla	EU692851.1	Corylus heterophylla clone c12 nitrate reductase gene, partial cds; and intron 3
Corylus_heterophylla	NC_031856	Corylus heterophylla chloroplast, complete genome
Corylus_heterophylla	FJ423710.1	Corylus heterophylla granule bound starch synthase (GBSSI) gene, exons 4, 5 and partial cds

Corylus_heterophylla	FJ423661.1	Corylus heterophylla atpB-rbcL intergenic spacer, partial sequence; and ribulose-1,5-bisphosphate carboxylase/oxygenase large subunit (rbcL) gene, partial cds; chloroplast
Corylus_heterophylla	AF254283.1	Corylus heterophylla clone 3D 5S ribosomal RNA non-transcribed spacer
Corylus_heterophylla	NC_031856	Corylus heterophylla chloroplast, complete genome
Corylus_heterophylla	AF081519	Corylus heterophylla internal transcribed spacer 1, 5.8S ribosomal RNA gene, and internal transcribed spacer 2, complete sequence
Corylus_heterophylla	KF419085	Corylus heterophylla trnL-trnF intergenic spacer, complete sequence; plastid
Corylus_heterophylla	FJ012031	Corylus heterophylla voucher Yoo s.n. tRNA-Leu (trnL) gene, partial sequence; chloroplast
Corylus_heterophylla	FJ011915	"Corylus heterophylla voucher Yoo s.n. ribosomal protein L16 (rpl16) gene, exons 1, 2 and partial cds; chloroplast"
Corylus_heterophylla_sutchuenensis	FJ011980.1	Corylus heterophylla var. sutchuenensis voucher MacAtrher-Tibet Expedition 1971 rps16 gene, intron; chloroplast
Corylus_heterophylla_sutchuenensis	FJ011855.1	Corylus heterophylla var. sutchuenensis voucher Tibet1971 PsbA (psbA) gene, partial cds; psbA-trnH intergenic spacer, complete sequence; and tRNA-His (trnH) gene, partial sequence; chloroplast
Corylus_heterophylla_sutchuenensis	FJ041251.1	Corylus heterophylla var. sutchuenensis voucher MacArthur-Tibet Expedition 1971 cytosolic phosphoglucose isomerase (pgiC) gene, exons 13 through 15 and partial cds
Corylus_heterophylla_sutchuenensis	FJ423711.1	Corylus heterophylla var. sutchuenensis granule bound starch synthase (GBSSI) gene, exons 4, 5 and partial cds
Corylus_heterophylla_sutchuenensis	FJ423662.1	Corylus heterophylla var. sutchuenensis atpB-rbcL intergenic spacer, partial sequence; and ribulose-1,5-bisphosphate carboxylase/oxygenase large subunit (rbcL) gene, partial cds; chloroplast
Corylus_heterophylla_sutchuenensis	AF254291.1	Corylus heterophylla var. sutchuenensis clone 2B 5S ribosomal RNA non-transcribed spacer
Corylus_heterophylla_sutchuenensis	AF297350	Corylus heterophylla var. sutchuensis clone Chet1ITS internal transcribed spacer 1, partial sequence; 5.8S ribosomal RNA gene, complete sequence; and internal transcribed spacer 2, partial sequence
Corylus_heterophylla_sutchuenensis	AF297380	Corylus heterophylla var. sutchuensis clone Chet1matK maturase K (matK) gene, partial cds; chloroplast gene for chloroplast product
Corylus_heterophylla_sutchuenensis	FJ012033	Corylus heterophylla var. sutchuenensis voucher MacArther-Tibet Expedition 1971 tRNA-Leu (trnL) gene, partial sequence; trnL-trnF intergenic spacer, complete sequence; and tRNA-Phe (trnF) gene, partial sequence; chloroplast
Corylus_heterophylla_sutchuenensis	FJ012033	Corylus heterophylla var. sutchuenensis voucher MacArther-Tibet Expedition 1971 tRNA-Leu (trnL) gene, partial sequence; trnL-trnF intergenic spacer, complete sequence; and tRNA-Phe (trnF) gene, partial sequence; chloroplast
Corylus_heterophylla_sutchuenensis	FJ011917	"Corylus heterophylla var. sutchuenensis voucher MacAtrher-Tibet Expedition 1971 ribosomal protein L16 (rpl16) gene, exons 1, 2 and partial cds; chloroplast"
Corylus_heterophylla_thunbergii	FJ011979.1	Corylus heterophylla var. thunbergii voucher Yoo s.n. rps16 gene, intron; chloroplast
Corylus_heterophylla_thunbergii	FJ011854.1	Corylus heterophylla var. thunbergii voucher Yoo s.n. PsbA (psbA) gene, partial cds; psbA-trnH intergenic spacer, complete sequence; and tRNA-His (trnH) gene, partial sequence; chloroplast
Corylus_heterophylla_thunbergii	FJ041250.1	Corylus heterophylla var. thunbergii voucher Yoo s.n. cytosolic phosphoglucose isomerase (pgiC) gene, exons 13, 14 and partial cds
Corylus_heterophylla_thunbergii	FJ423709.1	Corylus heterophylla var. thunbergii granule bound starch synthase (GBSSI) gene, exons 4, 5 and partial cds
Corylus_heterophylla_thunbergii	FJ423660.1	Corylus heterophylla var. thunbergii atpB-rbcL intergenic spacer, partial sequence; and ribulose-1,5-bisphosphate carboxylase/oxygenase large subunit (rbcL) gene, partial cds; chloroplast
Corylus_heterophylla_thunbergii	FJ011746	Corylus heterophylla var. thunbergii voucher Yoo s.n. 18S ribosomal RNA gene, partial sequence; internal transcribed spacer 1, 5.8S ribosomal RNA gene, and internal transcribed spacer 2, complete sequence; and 26S ribosomal RNA gene, partial sequence
Corylus_heterophylla_thunbergii	FJ011802	Corylus heterophylla var. thunbergii voucher Yoo s.n. maturaseK (matK) gene, partial cds; chloroplast
Corylus_heterophylla_thunbergii	FJ012032	Corylus heterophylla var. thunbergii voucher Yoo s.n. tRNA-Leu (trnL) gene and trnL-trnF intergenic spacer, partial sequence; chloroplast

Corylus_heterophylla_thunbergii	FJ012032	Corylus heterophylla var. thunbergii voucher Yoo s.n. tRNA-Leu (trnL) gene and trnL-trnF intergenic spacer, partial sequence; chloroplast
Corylus_heterophylla_thunbergii	FJ011916	"Corylus heterophylla var. thunbergii voucher Yoo s.n. ribosomal protein L16 (rpl16) gene, exons 1, 2 and partial cds; chloroplast"
Corylus_jacquemontii	KF418953.1	Corylus jacquemontii ribulose-1,5-bisphosphate carboxylase/oxygenase large subunit (rbcL) gene, partial cds; chloroplast
Corylus_jacquemontii	AF297390.1	Corylus jacquemontii clone Cjac1matK maturase K (matK) gene, partial cds; chloroplast gene for chloroplast product
Corylus_jacquemontii	KF418887.1	Corylus jacquemontii atpB-rbcL intergenic spacer, complete sequence; plastid
Corylus_jacquemontii	AF254317.1	Corylus jacquemontii clone 1F 5S ribosomal RNA non-transcribed spacer
Corylus_jacquemontii	AF297360.1	Corylus jacquemontii clone Cjac1ITS internal transcribed spacer 1, partial sequence; 5.8S ribosomal RNA gene, complete sequence; and internal transcribed spacer 2, partial sequence
Corylus_jacquemontii	KF419086	Corylus jacquemontii trnL-trnF intergenic spacer, complete sequence; plastid
Corylus_jacquemontii	KF418999	Corylus jacquemontii tRNA-Leu (trnL) gene, partial sequence; plastid
Corylus_maxima	KF418955.1	Corylus maxima ribulose-1,5-bisphosphate carboxylase/oxygenase large subunit (rbcL) gene, partial cds; chloroplast
Corylus_maxima	HE966583.1	Corylus maxima chloroplast DNA containing psbA-trnH IGS, specimen voucher MIB:ZPL:03535
Corylus_maxima	AF297367.1	Corylus maxima clone Cmax2matK maturase K (matK) gene, partial cds; chloroplast gene for chloroplast product
Corylus_maxima	KF418888.1	Corylus maxima atpB-rbcL intergenic spacer, complete sequence; plastid
Corylus_maxima	AJ784231.1	Corylus maxima partial 5S rRNA gene, NTS and partial 5S rRNA gene, specimen voucher Chase 6129 (K)
Corylus_maxima	AF297337.1	Corylus maxima clone Cmax2ITS internal transcribed spacer 1, partial sequence; 5.8S ribosomal RNA gene, complete sequence; and internal transcribed spacer 2, partial sequence
Corylus_maxima	KF419087	Corylus maxima trnL-trnF intergenic spacer, complete sequence; plastid
Corylus_maxima	KF419001	Corylus maxima tRNA-Leu (trnL) gene, partial sequence; plastid
Corylus_sieboldiana	FJ011981.1	Corylus sieboldiana voucher Yoo s.n. rps16 gene, intron; chloroplast
Corylus_sieboldiana	FJ011856.1	Corylus sieboldiana voucher Yoo s.n. PsbA (psbA) gene, partial cds; psbA-trnH intergenic spacer, complete sequence; and tRNA-His (trnH) gene, partial sequence; chloroplast
Corylus_sieboldiana	FJ041252.1	Corylus sieboldiana voucher Yoo s.n. cytosolic phosphoglucose isomerase (pgiC) gene, exons 13, 14 and partial cds
Corylus_sieboldiana	EU692848.1	Corylus sieboldiana clone c24 nitrate reductase gene, partial cds; and intron 3
Corylus_sieboldiana	FJ011804.1	Corylus sieboldiana voucher Yoo s.n. maturaseK (matK) gene, partial cds; chloroplast
Corylus_sieboldiana	FJ423712.1	Corylus sieboldiana granule bound starch synthase (GBSSI) gene, exons 4, 5 and partial cds
Corylus_sieboldiana	FJ423663.1	Corylus sieboldiana atpB-rbcL intergenic spacer, partial sequence; and ribulose-1,5-bisphosphate carboxylase/oxygenase large subunit (rbcL) gene, partial cds; chloroplast
Corylus_sieboldiana	AF254331.1	Corylus sieboldiana clone 1F 5S ribosomal RNA non-transcribed spacer
Corylus_sieboldiana	FJ011748.1	Corylus sieboldiana voucher Yoo s.n. internal transcribed spacer 1, partial sequence; 5.8S ribosomal RNA gene and internal transcribed spacer 2, complete sequence; and 26S ribosomal RNA gene, partial sequence
Corylus_sieboldiana	FJ012034	Corylus sieboldiana voucher Yoo s.n. tRNA-Leu (trnL) gene, partial sequence; chloroplast
Corylus_sieboldiana	FJ011918	"Corylus sieboldiana voucher Yoo s.n. ribosomal protein L16 (rpl16) gene, exon 2 and partial cds; chloroplast"

Corylus_sieboldiana_mandshurica	FJ011982.1	Corylus mandshurica voucher Yoo s.n. rps16 gene, intron; chloroplast
Corylus_sieboldiana_mandshurica	KF418954.1	Corylus mandshurica ribulose-1,5-bisphosphate carboxylase/oxygenase large subunit (rbcL) gene, partial cds; chloroplast
Corylus_sieboldiana_mandshurica	FJ011857.1	Corylus mandshurica voucher Yoo s.n. PsbA (psbA) gene, partial cds; psbA-trnH intergenic spacer, complete sequence; and tRNA-His (trnH) gene, partial sequence; chloroplast
Corylus_sieboldiana_mandshurica	FJ041253.1	Corylus mandshurica voucher Yoo s.n. cytosolic phosphoglucose isomerase (pgiC) gene, exons 13, 14 and partial cds
Corylus_sieboldiana_mandshurica	KF419041.1	Corylus mandshurica maturase K (matK) gene, partial cds; chloroplast
Corylus_sieboldiana_mandshurica	FJ423713.1	Corylus mandshurica granule bound starch synthase (GBSSI) gene, exons 4, 5 and partial cds
Corylus_sieboldiana_mandshurica	FJ423664.1	Corylus mandshurica atpB-rbcL intergenic spacer, partial sequence; and ribulose-1,5-bisphosphate carboxylase/oxygenase large subunit (rbcL) gene, partial cds; chloroplast
Corylus_sieboldiana_mandshurica	FJ011749.1	Corylus mandshurica voucher Yoo s.n. 18S ribosomal RNA gene, partial sequence; internal transcribed spacer 1, 5.8S ribosomal RNA gene, and internal transcribed spacer 2, complete sequence; and 26S ribosomal RNA gene, partial sequence
Corylus_sieboldiana_mandshurica	AF254336	Corylus sieboldiana var. mandshurica clone 1D 5S ribosomal RNA non-transcribed spacer
Corylus_sieboldiana_mandshurica	FJ012035	Corylus mandshurica voucher Yoo s.n. tRNA-Leu (trnL) gene, partial sequence; trnL-trnF intergenic spacer, complete sequence; and tRNA-Phe (trnF) gene, partial sequence; chloroplast
Corylus_sieboldiana_mandshurica	FJ012035	Corylus mandshurica voucher Yoo s.n. tRNA-Leu (trnL) gene, partial sequence; trnL-trnF intergenic spacer, complete sequence; and tRNA-Phe (trnF) gene, partial sequence; chloroplast
Corylus_sieboldiana_mandshurica	FJ011919	"Corylus mandshurica voucher Yoo s.n. ribosomal protein L16 (rpl16) gene, exons 1, 2 and partial cds; chloroplast"
Corylus_yunnanensis	FJ011984.1	Corylus yunnanensis voucher Tibet953 rps16 gene, intron; chloroplast
Corylus_yunnanensis	FJ011859.1	Corylus yunnanensis voucher Tibet953 PsbA (psbA) gene, partial cds; psbA-trnH intergenic spacer, complete sequence; and tRNA-His (trnH) gene, partial sequence; chloroplast
Corylus_yunnanensis	FJ041255.1	Corylus yunnanensis voucher Tibet953 cytosolic phosphoglucose isomerase (pgiC) gene, exons 13 through 15 and partial cds
Corylus_yunnanensis	FJ011807.1	Corylus yunnanensis voucher Tibet953 maturaseK (matK) gene, partial cds; chloroplast
Corylus_yunnanensis	FJ423715.1	Corylus yunnanensis granule bound starch synthase (GBSSI) gene, exons 4, 5 and partial cds
Corylus_yunnanensis	FJ423666.1	Corylus yunnanensis atpB-rbcL intergenic spacer, partial sequence; and ribulose-1,5-bisphosphate carboxylase/oxygenase large subunit (rbcL) gene, partial cds; chloroplast
Corylus_yunnanensis	AF254285.1	Corylus heterophylla var. yunnanensis clone 1B 5S ribosomal RNA non-transcribed spacer
Corylus_yunnanensis	AF297352.1	Corylus heterophylla var. yunnanensis clone Chet3ITS internal transcribed spacer 1, partial sequence; 5.8S ribosomal RNA gene, complete sequence; and internal transcribed spacer 2, partial sequence
Corylus_yunnanensis	FJ012037	Corylus yunnanensis voucher Tibet953 tRNA-Leu (trnL) gene, partial sequence; trnL-trnF intergenic spacer, complete sequence; and tRNA-Phe (trnF) gene, partial sequence; chloroplast
Corylus_yunnanensis	FJ012037	Corylus yunnanensis voucher Tibet953 tRNA-Leu (trnL) gene, partial sequence; trnL-trnF intergenic spacer, complete sequence; and tRNA-Phe (trnF) gene, partial sequence; chloroplast
Corylus_yunnanensis	FJ011921	"Corylus yunnanensis voucher Tibet953 ribosomal protein L16 (rpl16) gene, exons 1, 2 and partial cds; chloroplast"
Ostrya_rehderiana	EU692867	Ostrya rehderiana clone c44 nitrate reductase gene, partial cds; and intron 3
Ostrya_rehderiana	FJ423719	Ostrya rehderiana granule bound starch synthase (GBSSI) gene, exons 4, 5 and partial cds
Ostrya_rehderiana	NC_028349	Ostrya rehderiana chloroplast, complete genome

Ostrya_rehderiana	NC_028349	Ostrya rehderiana chloroplast, complete genome
Ostrya_rehderiana	NC_028349	Ostrya rehderiana chloroplast, complete genome
Ostrya_rehderiana	FJ011989	Ostrya rehderiana voucher Fu99001 rps16 gene, intron; chloroplast
Ostrya_rehderiana	FJ041262	Ostrya rehderiana voucher Wen97_24 cytosolic phosphoglucose isomerase (pgiC) gene, exons 13 through 15 and partial cds
Ostrya_rehderiana	KX305960	Ostrya rehderiana internal transcribed spacer 1, partial sequence; 5.8S ribosomal RNA gene, complete sequence; and internal transcribed spacer 2, partial sequence
Ostrya_rehderiana	FJ423670	Ostrya rehderiana atpB-rbcL intergenic spacer, partial sequence; and ribulose-1,5-bisphosphate carboxylase/oxygenase large subunit (rbcL) gene, partial cds; chloroplast
Ostrya_rehderiana	NC_028349	Ostrya rehderiana chloroplast, complete genome
Ostrya_rehderiana	NC_028349	Ostrya rehderiana chloroplast, complete genome

Table S4 Results and parameters of BioGeoBEARS analyses. Best fitting models for each set of analyses is highlighted in yellow.

Tree	Restriction	Model	LnL	numparams	d	e	j	AICc	AICc_wt
Nuclear	None	DEC	-53.9	2	0.015	0.016	0	112.7	0.015
Nuclear	None	DEC+J	-48.22	3	0.0083	1.00E-12	0.11	104.4	0.96
Nuclear	None	DIVALIKE	-55.33	2	0.018	1.00E-12	0	115.6	0.0037
Nuclear	None	DIVALIKE+J	-52.4	3	0.012	3.60E-09	0.062	112.8	0.015
Nuclear	None	BAYAREALIKE	-57.97	2	0.021	0.17	0	120.9	0.0003
Nuclear	None	BAYAREALIKE+J	-54.66	3	0.0093	1.00E-07	0.082	117.3	0.0015
Nuclear	Areas allowed	DEC	-54.63	2	0.05	0.061	0	114.2	1.00E-04
Nuclear	Areas allowed	DEC+J	-44.3	3	0.019	1.00E-12	0.15	96.61	0.96
Nuclear	Areas allowed	DIVALIKE	-54.91	2	0.055	0.043	0	114.7	1.00E-04
Nuclear	Areas allowed	DIVALIKE+J	-47.55	3	0.026	1.00E-12	0.071	103.1	0.037
Nuclear	Areas allowed	BAYAREALIKE	-58.61	2	0.065	0.16	0	122.2	2.70E-06
Nuclear	Areas allowed	BAYAREALIKE+J	-50.05	3	0.018	1.00E-07	0.095	108.1	0.0031
Plastid	None	DEC	-53.5	2	0.011	1.40E-02	0	111.9	0.0015
Plastid	None	DEC+J	-50.38	3	0.0083	1.00E-12	0.027	108.8	0.0074
Plastid	None	DIVALIKE	-55.99	2	0.014	1.30E-02	0	116.9	0.0001
Plastid	None	DIVALIKE+J	-52.75	3	0.0099	1.00E-12	0.023	113.5	0.0007
Plastid	None	BAYAREALIKE	-49.89	2	0.01	0.12	0	104.7	0.056
Plastid	None	BAYAREALIKE+J	-45.54	3	0.0058	1.00E-07	0.041	99.09	0.93
Plastid	Areas allowed	DEC	-53.13	2	0.037	0.043	0	111.2	4.30E-05
Plastid	Areas allowed	DEC+J	-46.38	3	0.021	1.00E-12	0.039	100.8	0.008
Plastid	Areas allowed	DIVALIKE	-54.99	2	0.042	0.043	0	114.9	6.70E-06
Plastid	Areas allowed	DIVALIKE+J	-48.25	3	0.024	1.00E-12	0.031	104.5	0.0012
Plastid	Areas allowed	BAYAREALIKE	-51.68	2	0.032	0.1	0	108.3	0.0002
Plastid	Areas allowed	BAYAREALIKE+J	-41.55	3	0.013	1.00E-07	0.041	91.11	0.99

Appendix S2 Gene trees inferred with RAxML. Numbers at nodes represent bootstrap support (100 replicates). A scale bar is shown for each tree and indicates the mean number of nucleotide substitutions per site on the respective branch.

RAxML_bipartitions.5s.fa

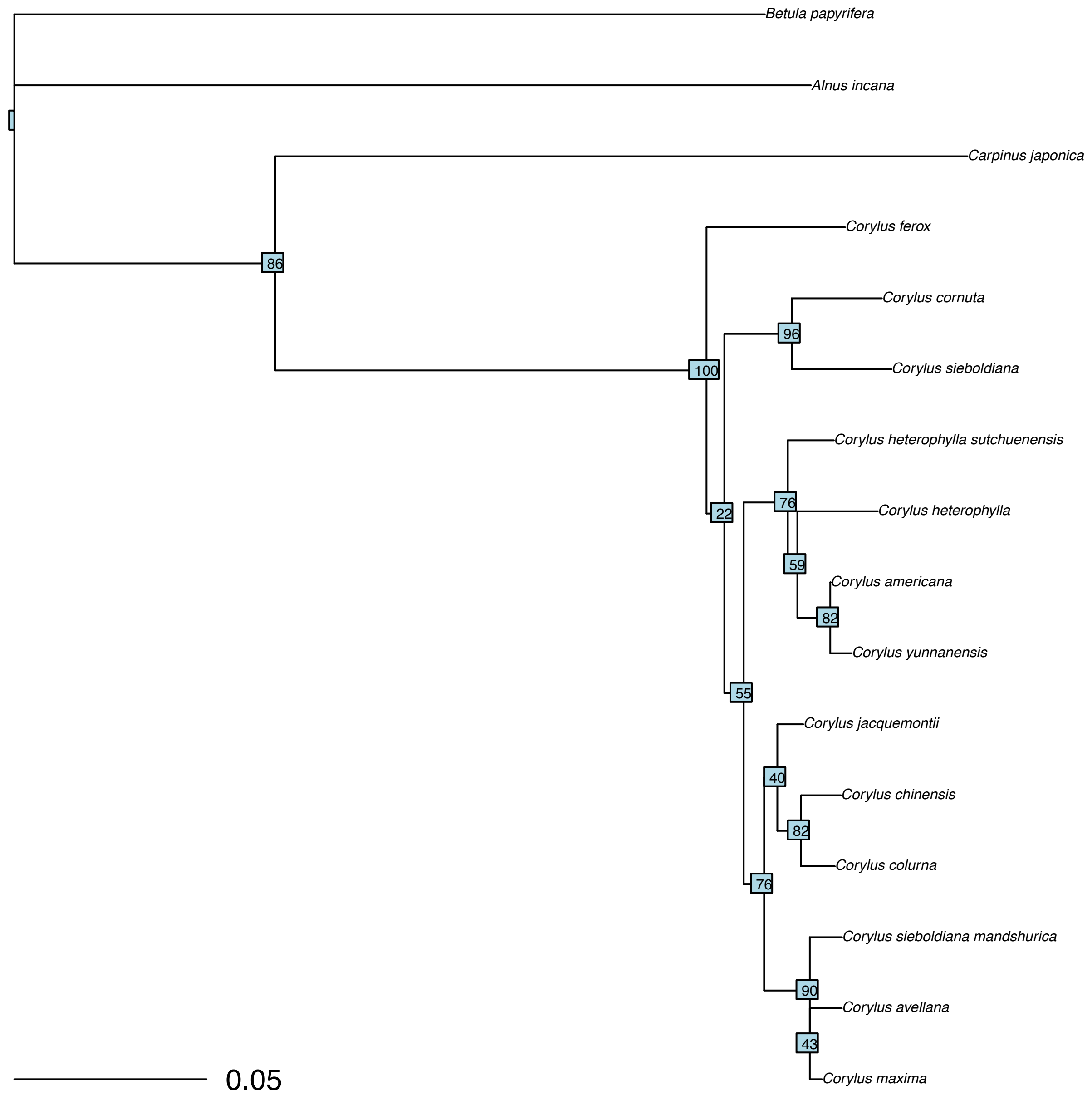

RAxML_bipartitions.atpB_rbcl.fa

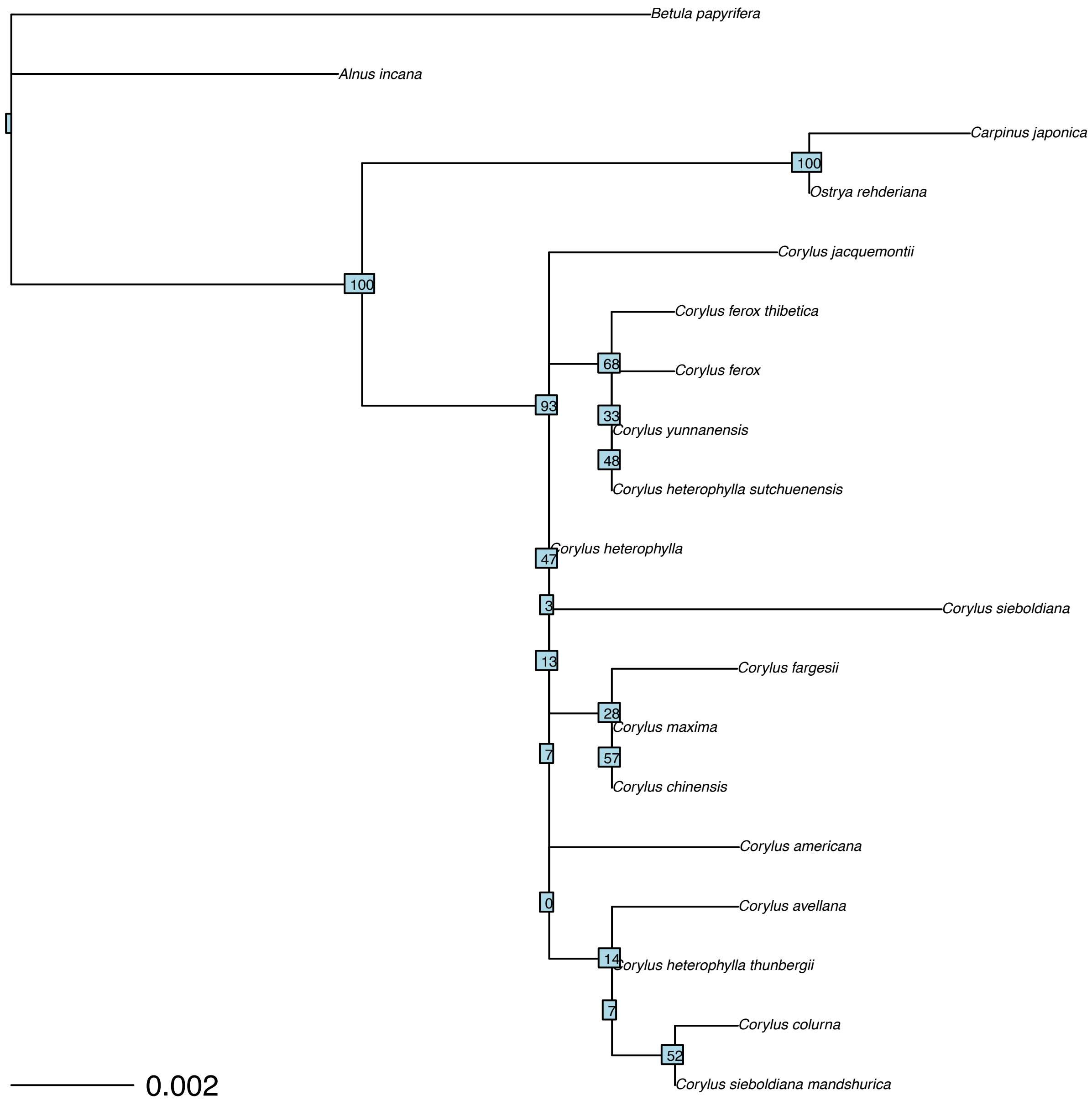

RAxML_bipartitions.gbssi.fa

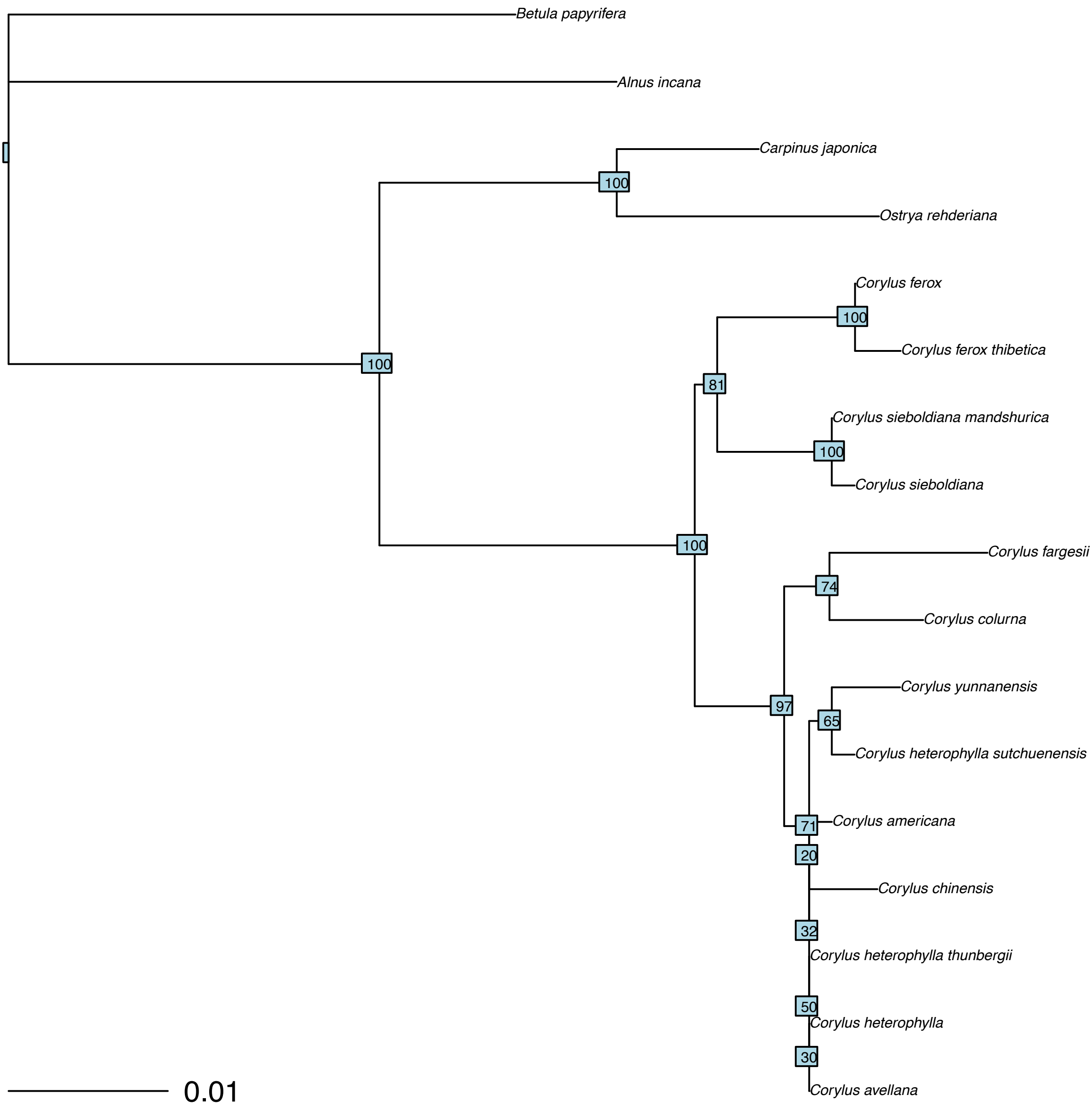

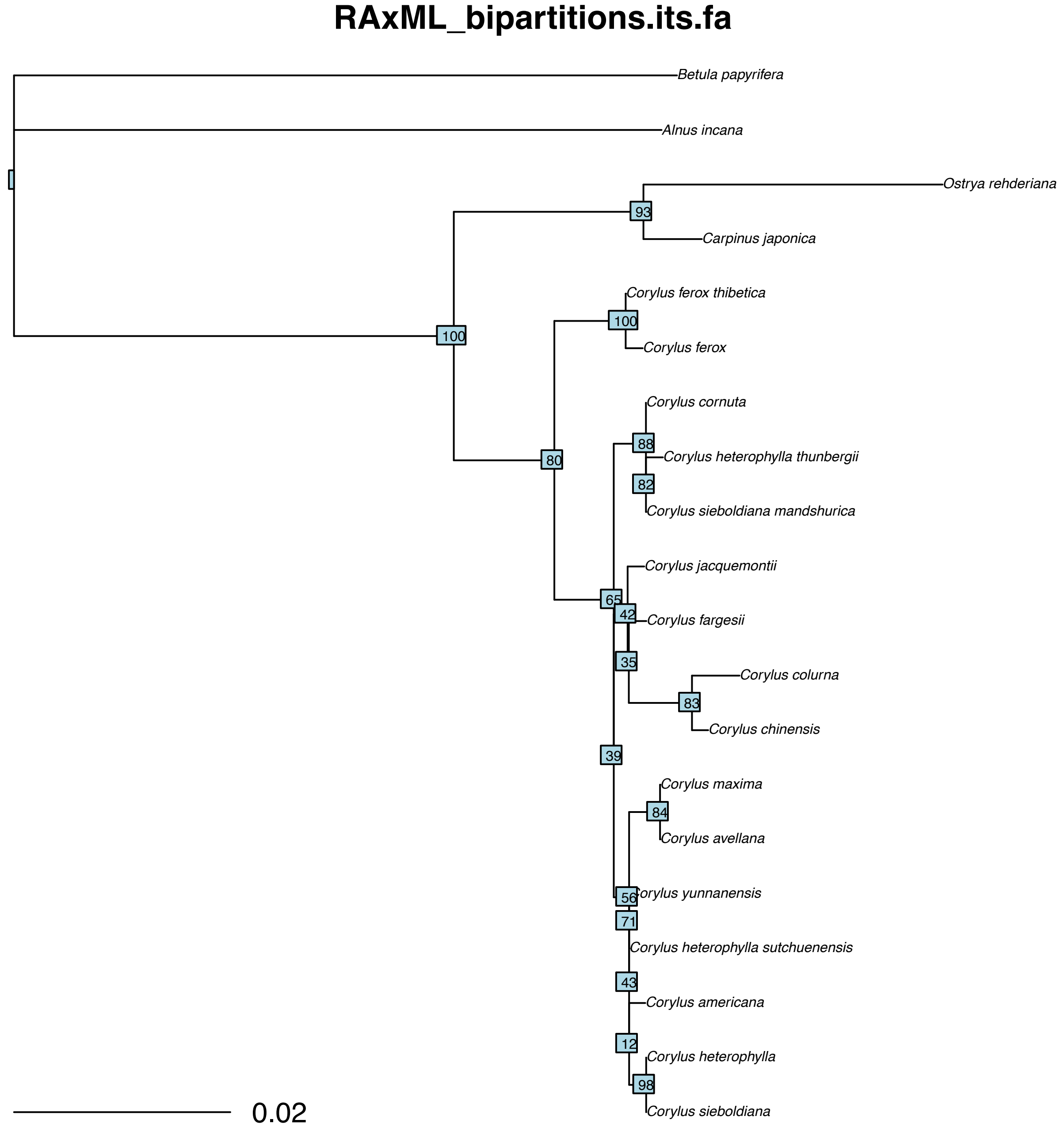
RAxML_bipartitions.its.fa
Betula papyrifera
Alnus incana
Ostrya rehderiana
93
Carpinus japonica
Corylus ferox thibetica
100
Corylus ferox
100
Corylus cornuta
88
Corylus heterophylla thunbergii
82
Corylus sieboldiana mandshurica
80
Corylus jacquemontii
65
42
Corylus fargesii
35
Corylus colurna
83
Corylus chinensis
39
Corylus maxima
84
Corylus avellana
56
Corylus yunnanensis
71
Corylus heterophylla sutchuenensis
43
Corylus americana
12
Corylus heterophylla
98
Corylus sieboldiana
0.02

RAxML_bipartitions.matk.fa

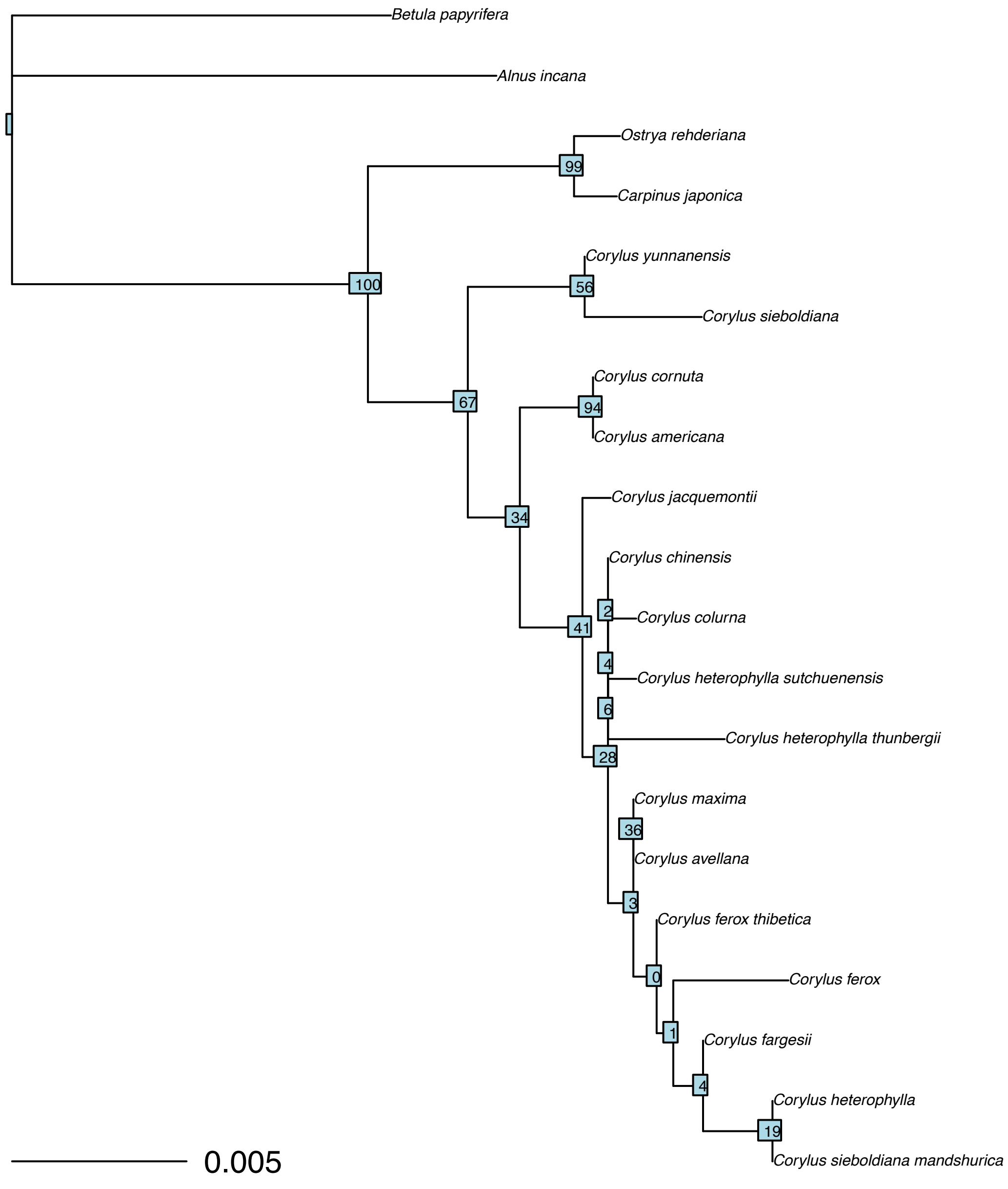

RAxML_bipartitions.pgic.fa

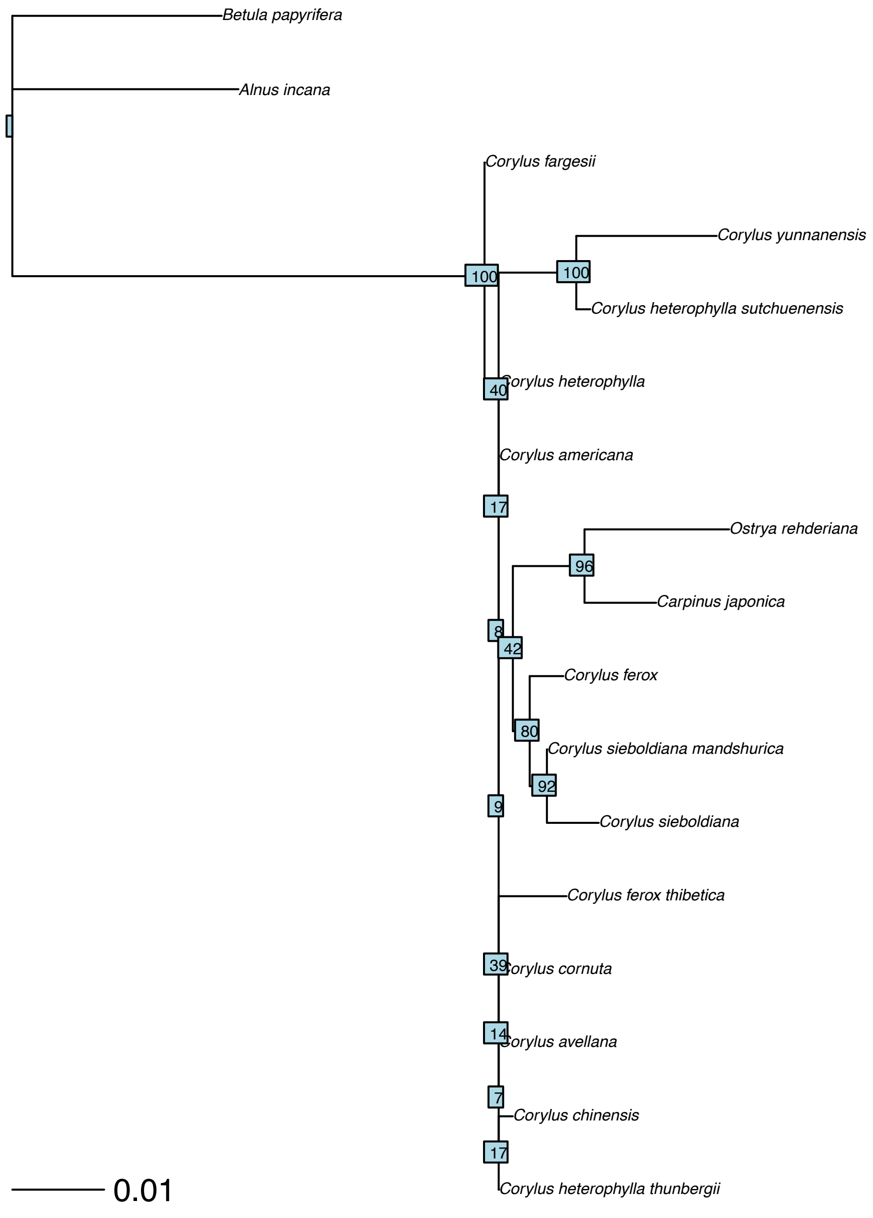

RAxML_bipartitions.psba.fa

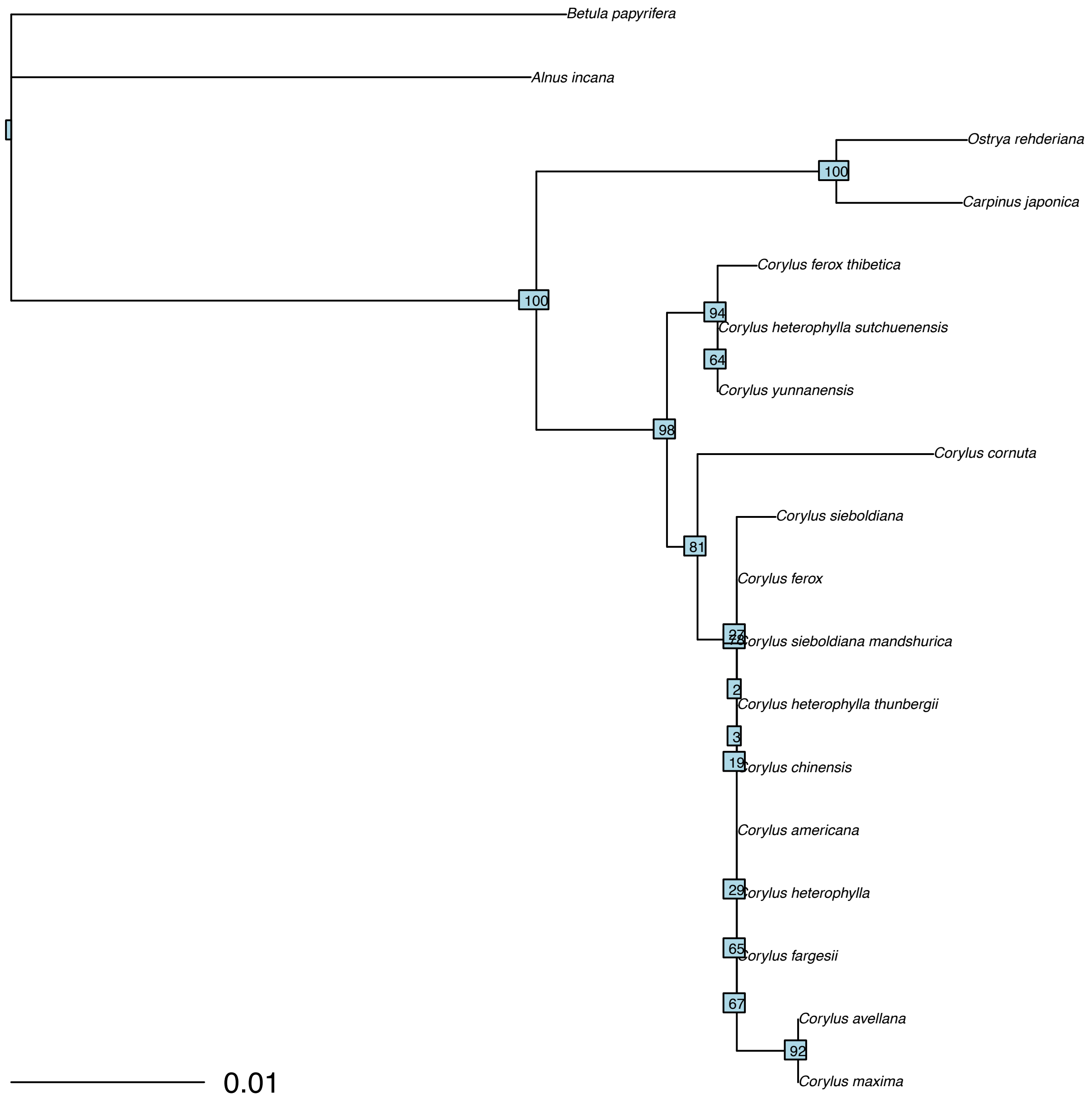

RAxML_bipartitions.rbcl.fa

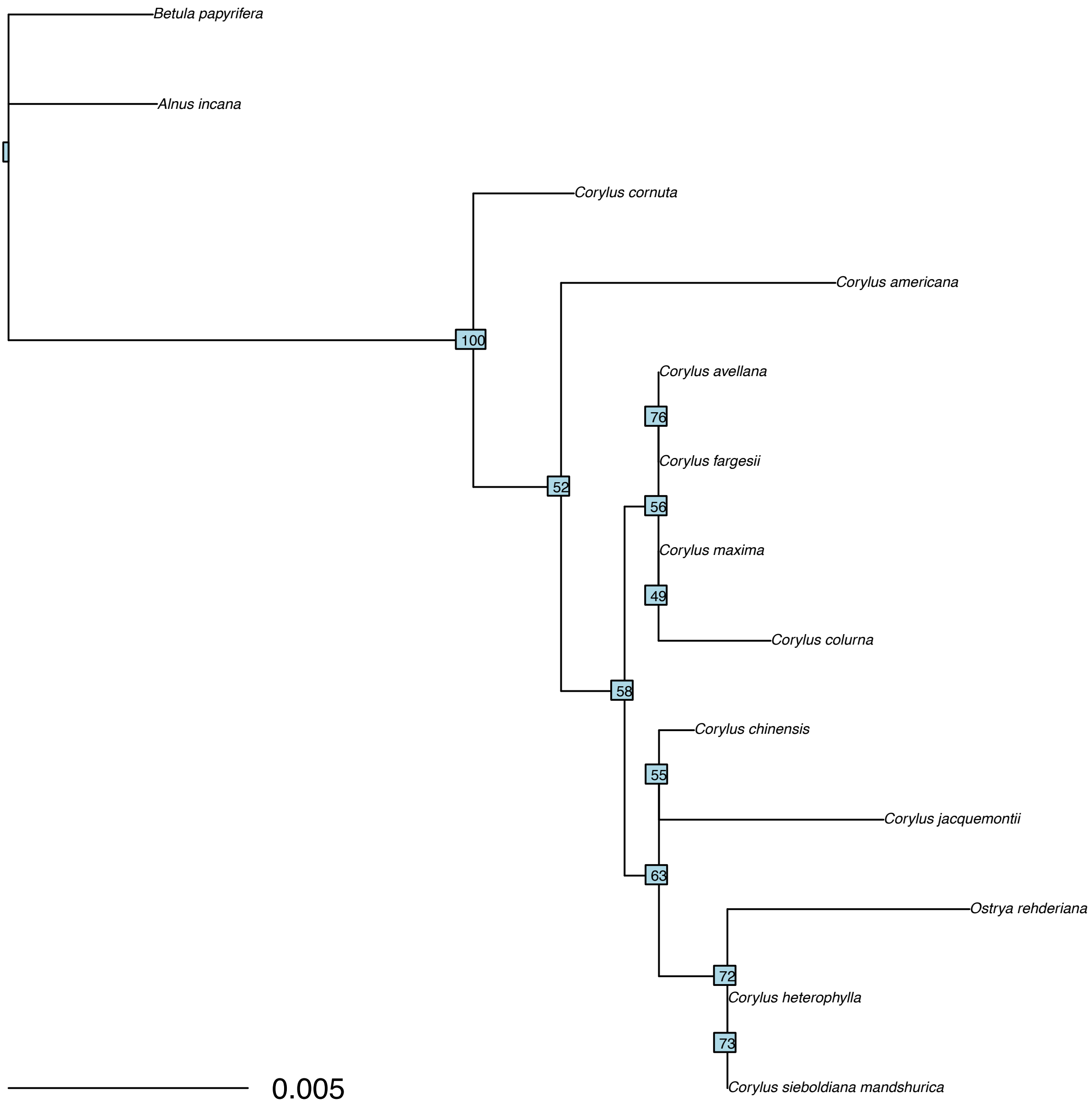

RAxML_bipartitions.rps16.fa

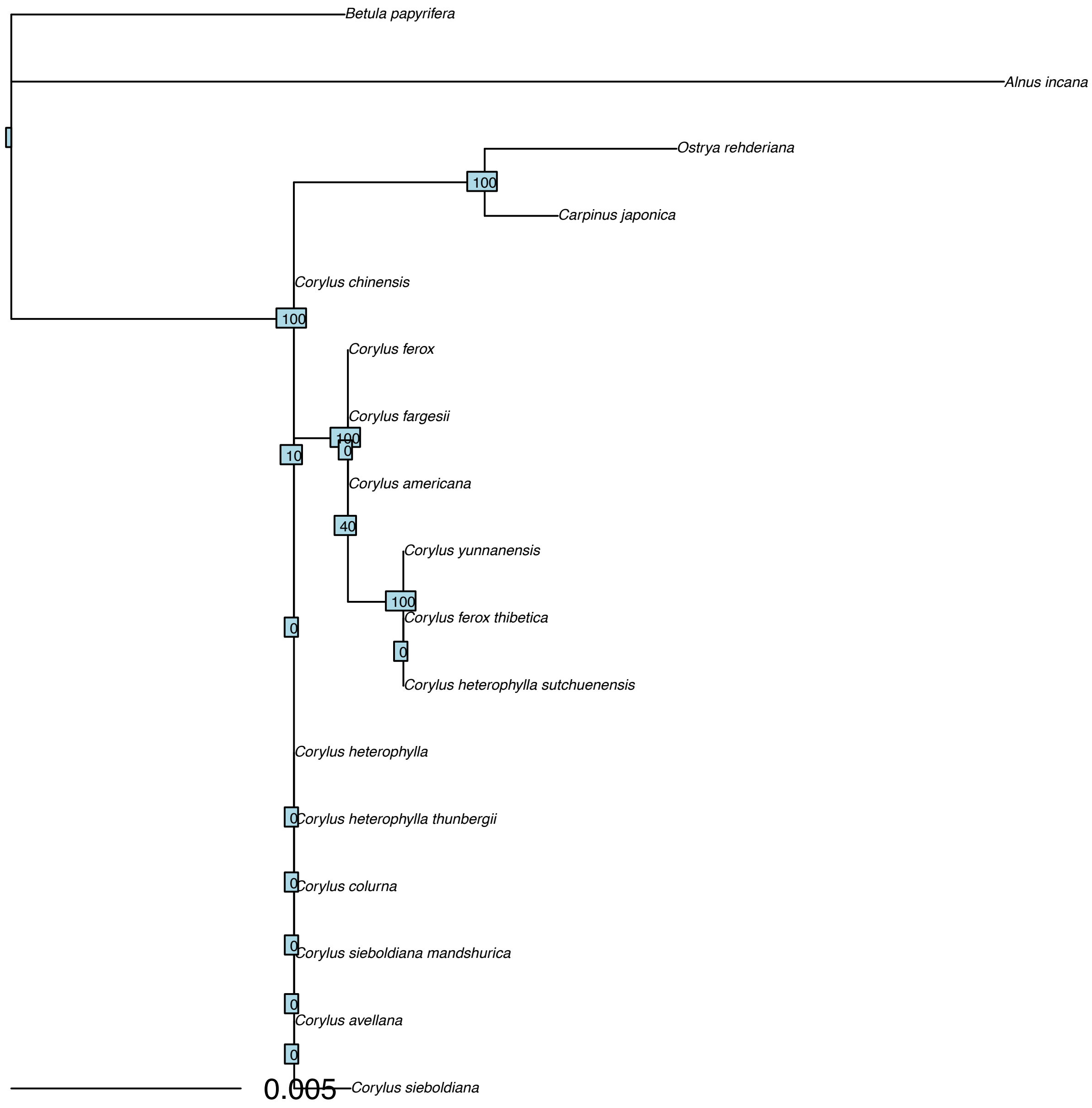

RAxML_bipartitions.trnl_trnf.fa

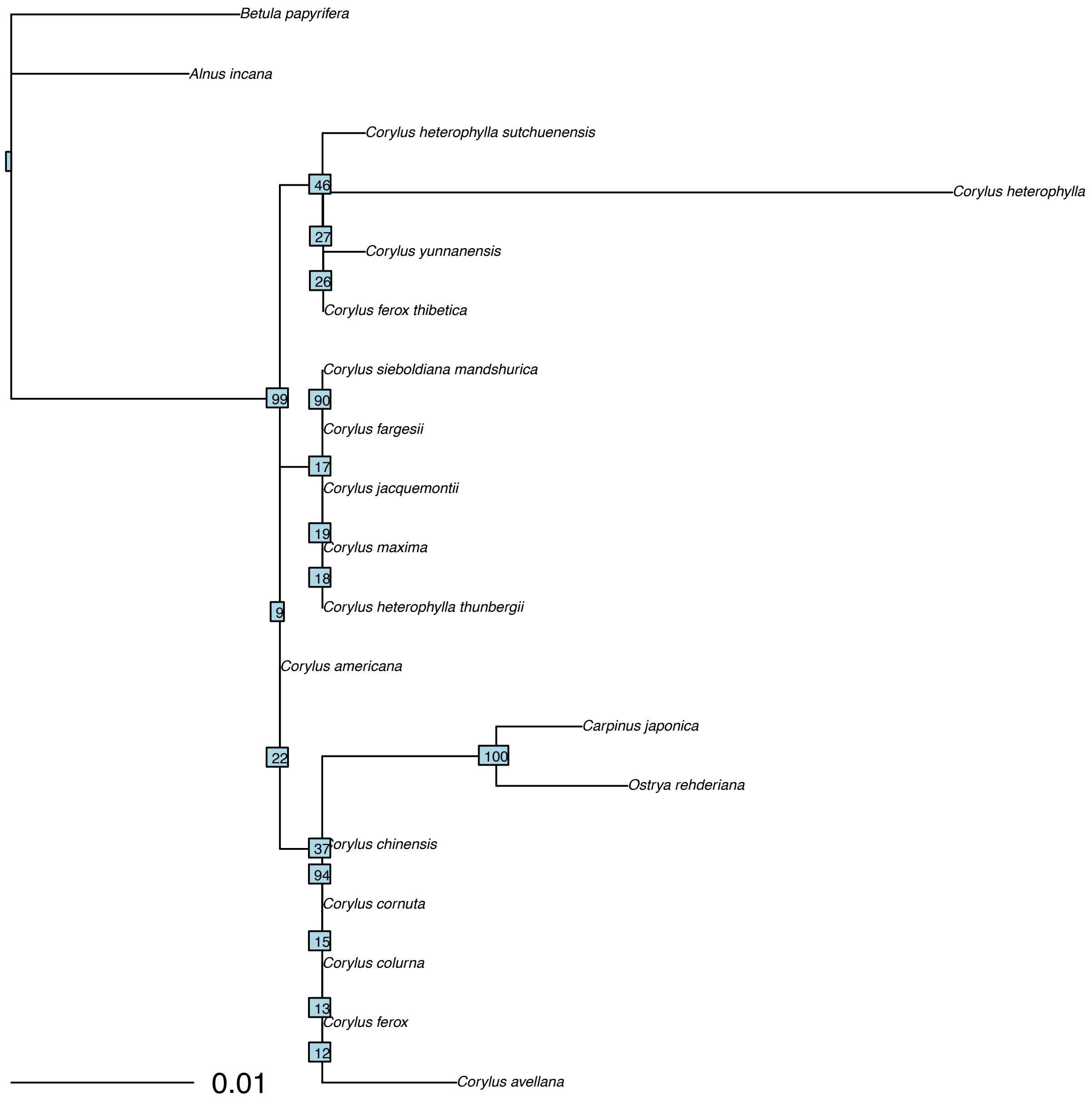

RAxML_bipartitions.trnl.fa

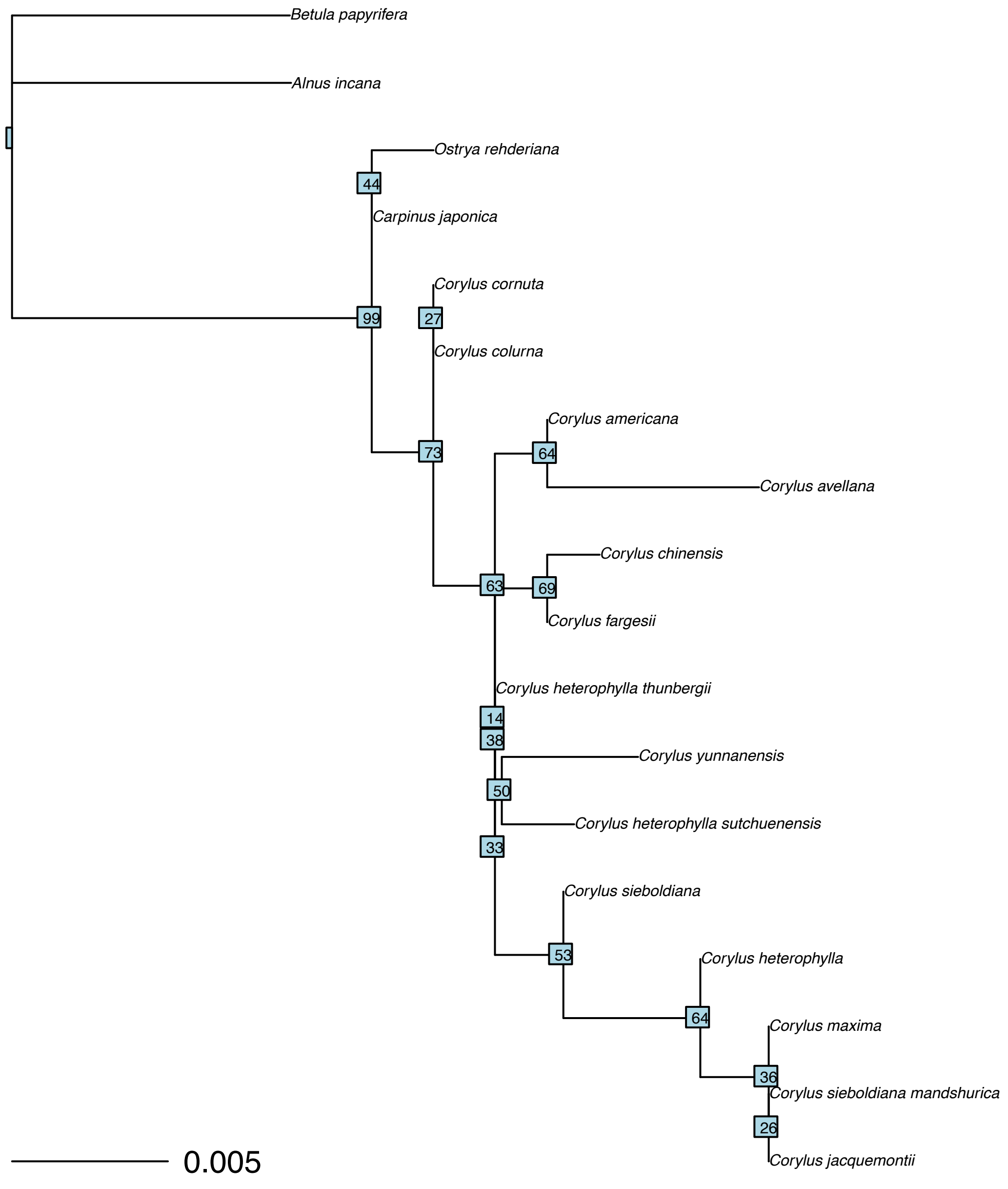

RAxML_bipartitions.nit_red.fa

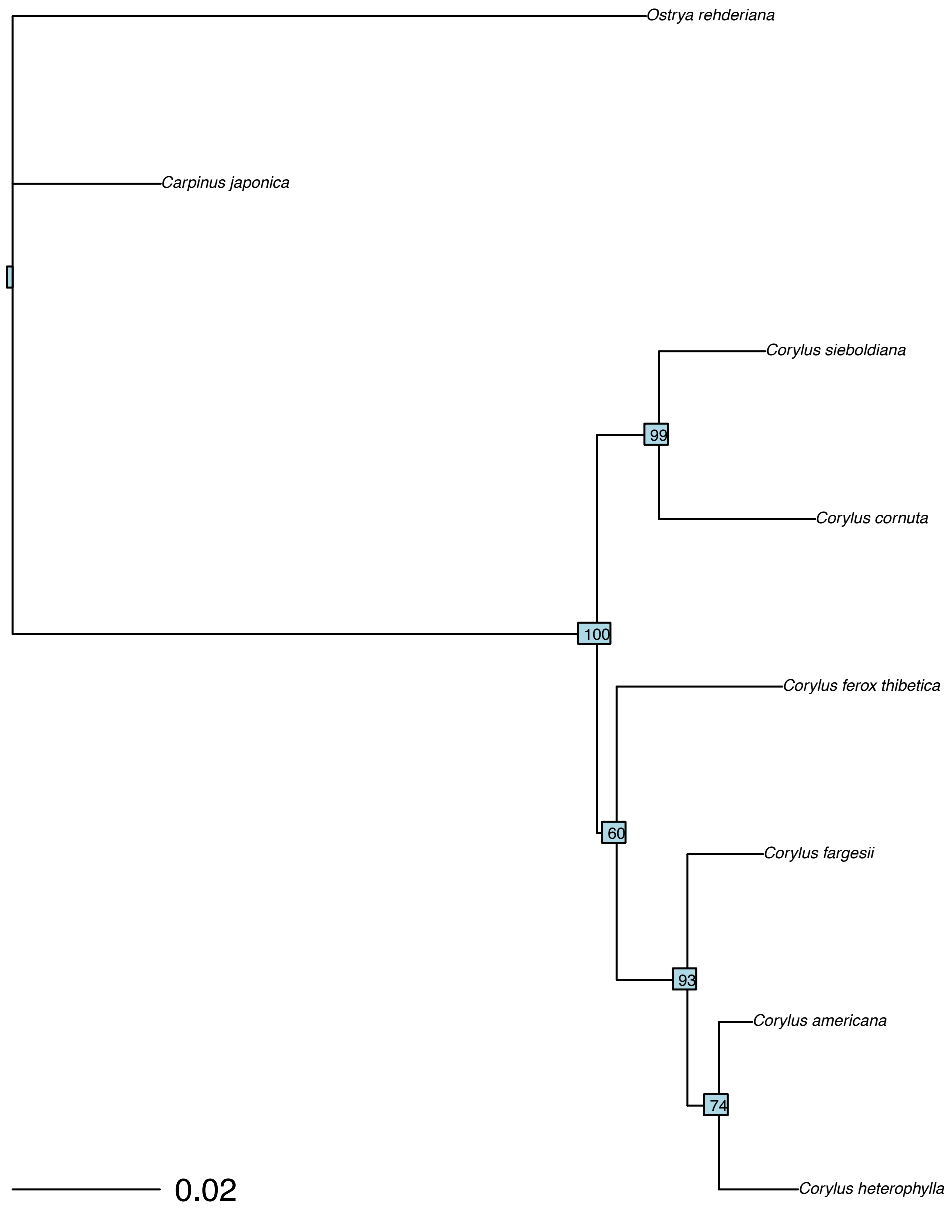

RAxML_bipartitions.rpl16.fa

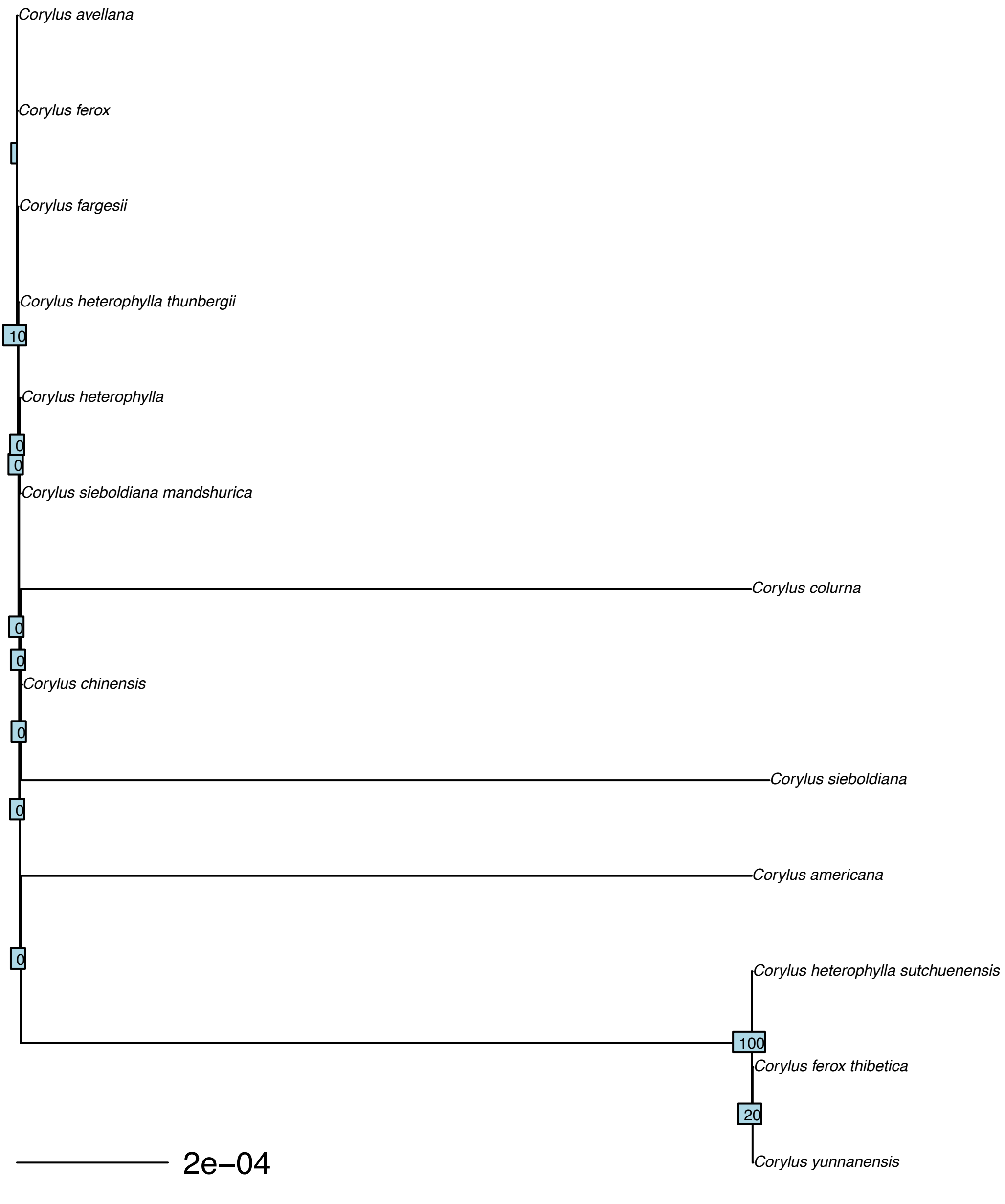

Supplement: Supplementary file 1 — Supplementary Materials [file 41598_2019_52403_MOESM1_ESM.pdf]
